# The impact of information about tobacco-related reproductive vs. general health risks on South Indian women’s tobacco use decisions

**DOI:** 10.1017/ehs.2020.61

**Published:** 2020-11-20

**Authors:** Caitlyn D. Placek, Renee E. Magnan, Vijaya Srinivas, Poornima Jaykrishna, Kavitha Ravi, Anisa Khan, Purnima Madhivanan, Edward H. Hagen

**Affiliations:** 1 Department of Anthropology, Ball State University, Muncie, Indiana, USA; 2 Department of Psychology, Washington State University, Vancouver, Washington, USA; 3 Public Health Research Institute of India, Mysore, Karnataka, India; 4 Department of Health Promotion Sciences, Mel & Enid Zuckerman College of Public Health, University of Arizona, Tucson, Arizona, USA; 5 Division of Infectious Diseases, College of Medicine, University of Arizona, Tucson, Arizona, USA; 6 Department of Family and Community Medicine, College of Medicine, University of Arizona, Tucson, Arizona, USA; 7 Department of Anthropology, Washington State University, Vancouver, Washington, USA

**Keywords:** tobacco control, randomized controlled trial, reproductive health, India, evolutionary theory

## Abstract

Smokeless tobacco use among Indian women is increasing despite prevention efforts. Evolutionary theories suggest that reproductive-aged women should be more concerned about immediate threats to reproduction than threats to survival occurring late in life. This study therefore compared an anti-tobacco intervention that emphasized near-term reproductive harms to one involving general harms occurring later in life. Scheduled Tribal women (*N* = 92) from Karnataka, India participated in this study. At baseline, women reported tobacco use and knowledge of harms, provided a saliva sample to assess use, and randomly viewed either a general harms presentation (GHP) or reproductive harms presentation (RHP). At followup, women reported their use, knowledge of harms and intentions to quit, and provided another saliva sample. At baseline, participants were aware of general harms but not reproductive harms. Both interventions increased knowledge of harms. Women in the RHP condition did not list more harms than women in the GHP condition, however, and the RHP was not more effective in reducing tobacco use than the GHP. In the RHP condition fetal health was particularly salient. In the GHP condition, oral health was highly salient, aligning with the local disease ecology and research on tobacco use and attractiveness.

## Media summary

Providing information on tobacco’s reproductive harms might reduce tobacco use among Scheduled Tribal women in India.

## Introduction

Owing to a combination of population growth, aging populations and increases in tobacco use, most tobacco-related deaths are projected to occur in low- and middle-income countries (LMICs) (Reitsma et al., 2017). One encouraging statistic is that whereas the prevalence of tobacco use among men in LMICs and high-income countries (HIC) is similarly high (about 30%), the prevalence of tobacco use among women in most LMICs is currently much lower than that in women in HICs (about 4% vs. 17%; Ng et al., 2014). Yet low use among women in LMICs makes this demographic a tempting target for tobacco companies, and existing health behaviour change initiatives in low income groups have only limited effectiveness (Bull, Dombrowski, McCleary, & Johnston, 2014). There is therefore an urgent need to develop public health initiatives to maintain low rates of tobacco use among women in LMICs.

Maintaining a low rate of tobacco use requires understanding why it is low to begin with. Gender inequality is a popular explanation for the low prevalence of female tobacco use in LMICs. Gender inequality is defined differently by different researchers, but generally refers to restrictions on female decision-making, access to education, political power and income (Lorber, 2012), which are linked to gender disparities in health outcomes (Heise et al., 2019). According to this view, cross-national sex differences in smoking prevalence rates are explained by cross-national differences in gender inequality, including the degree to which men and women adhere to traditional gender roles that discourage female tobacco use, encourage greater rebelliousness among men and leave women with less autonomy and money to acquire tobacco (Hitchman & Fong, 2011; Pathania, 2011).

Women in LMICs differ from those in HICs in a number of dimensions beyond those usually ascribed to gender inequality, however, many of them related to reproduction. Specifically, women in LMICs often get married at an earlier age, have an earlier age at first birth, and have higher total fertility rates than women in HICs (United Nations, 2015). From an evolutionary perspective, reproductive-aged women, and particularly pregnant women, evolved to avoid the consumption of toxic substances, such as tobacco, that could disrupt pregnancy and fetal development (‘maternal–fetal protection hypothesis’; Hook, 1978; Profet, 1995). Because tobacco is a toxic plant, adaptations for maternal–fetal protection could limit tobacco consumption during pregnancy and among reproductive-aged women more generally (Hagen, Roulette, & Sullivan, 2013). Women have reported aversions towards tobacco during periods of heightened vulnerability (Tierson, Olsen, & Hook, 1985), such as during the first trimester when aspects of the immune system shift to support implantation, placentation and organogenesis (Mor & Cardenas, 2010; Pazos, Sperling, Moran, & Kraus, 2012). Thus, in populations in which women marry and have children at an early age, and have relatively high total fertility, fetal protection mechanisms might deter the transition to tobacco use. In support, Hagen et al. (2013) found that, controlling for multiple indices of gender inequality, higher total fertility rates were negatively associated with the prevalence of female smoking across nations.

Evolutionary theories of cultural transmission suggest that the costs of individual learning, such as accidentally eating a toxic substance, will favour social learning strategies (Rogers, 1988). Women should consequently not only experience physiological aversions to toxic substances, but also be motivated to learn which substances are deemed harmful during pregnancy. Studies confirm that women rely heavily on social learning to navigate the avoidance of toxic foods and substances that can disrupt fetal development during pregnancy (Henrich & Henrich, 2010; Placek, Madhivanan, & Hagen, 2017). In addition, peer-to-peer transmission of tobacco harms is an effective strategy in reducing use because peers are less intimidating than physicians (Secker-Walker et al., 2000; Williams et al., 2011).

Unfortunately, tobacco is not always viewed as harmful by women in LMICs, and is even thought to provide benefits, such as increasing energy (Placek et al., 2019). Part of the problem is that tobacco control programmes typically emphasize health-related harms that occur late in life, yet evolutionary theory suggests that individuals should discount costs and benefits that will occur later in life, a phenomenon termed temporal discounting. In humans, there is substantial evidence that adverse conditions such as disasters and low socioeconomic status increase temporal discounting, and such discounting would have increased fitness (for review and commentary, see Pepper & Nettle, 2017). Public health interventions that emphasize more immediate harms to fitness might therefore be more effective at changing behaviours than those that emphasize harms occurring late in life (Saad & Peng, 2006).

In India, one of the largest LMICs with a population of about 1.3 billion, women’s use of smokeless tobacco is increasing (Sinha et al., 2015). In some regions of India, rates of smokeless tobacco use among women is as high as 49% (P. C. Gupta & Ray, 2003; Placek et al., 2019), and women consume tobacco and *paan* (an areca nut preparation that often contains tobacco) during pregnancy and throughout their reproductive years (P. C. Gupta & Ray, 2003; Krisshna, 1978; Placek et al., 2019). These alarming rates are probably due, in part, to local perceptions that smokeless tobacco is less harmful than cigarettes (Sinha et al., 2015). Additionally, although all forms of tobacco can have detrimental effects on reproductive health, anti-smoking campaigns and graphic warnings in India usually target general health concerns (Raute, Pednekar, & Gupta, 2009), such as lung and oral cancer, that occur late in life. Further, tobacco control campaigns in India usually target pre-reproductive adolescents because, similar to other regions of the world, tobacco use typically onsets during this period (Arora et al., 2010; Shrivastav, Nazar, Stigler, & Arora, 2012). Although these interventions often emphasize both short- and long-term consequences of tobacco use, adolescents tend view the near-term harms of tobacco as being minimal and discount the long-term harms of use (Mishra et al., 2005). Tobacco control efforts also tend to be gender-neutral even though Indian men and women differ in the types of tobacco products they prefer (Amos, Greaves, Nichter, & Bloch, 2011; Nichter & Cartwright, 1991; Placek et al., 2019). Finally, the recent change in India from a symbolic warning label (a scorpion) to graphic warning labels (graphic images of cancer of the mouth, jaw or neck) was not associated with any improvement in health-related outcomes, such as awareness of the labels, thoughts about the harms of tobacco use or reduced tobacco use (Gravely et al., 2016).

A meta-analysis of health behaviour change interventions for diet, physical activity and tobacco use in low-income groups found only small positive effects, potentially exacerbating health inequalities (Bull et al., 2014). These factors highlight a need to test and implement improved tobacco prevention and cessation programmes that target the types of tobacco Indian women use with a messaging strategy that is specific to their immediate health concerns.

## Study aims

In response to the increasing prevalence of tobacco use among reproductive-aged women in India, the current study drew upon two evolutionary considerations to devise and test an anti-tobacco messaging strategy. According to relevance theory (Wilson & Sperber, 2004) and the fetal protection model (Hagen, Garfield, & Sullivan, 2016), this demographic should, first, be especially interested in learning about and avoiding teratogenic substances (fetal protection), which, second, pose immediate rather than long-delayed risks (temporal discounting), further increasing the relevance. We therefore aimed to compare a standard anti-tobacco messaging strategy about general health risks that tend to occur late in life with one that emphasized reproductive harms specific to women that tend to occur earlier in life.

We specifically predicted that reproductive-aged women in a disadvantaged population exposed to information about the near-term reproductive harms of tobacco use would be more likely to (1) reduce tobacco use, (2) increase knowledge of tobacco harms, (3) report more intentions to quit and (4) be more likely to share this information with others compared with women exposed to information about generic harms of tobacco use that are not specific to reproductive-aged women and tend to occur far in the future.

## Methods

### Study population

The adverse health consequences associated with tobacco use in India vary according to caste categories and socioeconomic status. Indigenous populations, nationally recognized as Scheduled Tribes, or Adivasis, are considered the most marginalized group in India with the greatest health disparities (Indian Institute of Population Sciences, 2000). Scheduled Tribes tend to report higher rates of tobacco use compared with other castes (Rani, 2003; Subramanian, Nandy, Kelly, Gordon, & Davey Smith, 2004; Subramanian, Smith, & Subramanyam, 2006), which probably helps explain why Scheduled Tribes also suffer from high rates of oral submucous fibrosis, oral cancer and hypertension, as well as other oral and cardiovascular diseases, all of which are linked to smokeless tobacco use (Deepa, Jose, & Prabhu, 2013; Deo, Pawar, Kanetkar, & Kakade, 2017; P. C. Gupta, Sinor, Bhonsle, Pawar, & Mehta, 1998; Mukhopadhyay, 2006). India also ranks among the highest in oral cancer rates among women (Mohan & Lando, 2016). Furthermore, smokeless tobacco use is strongly associated with still-births among Indian women (P. C. Gupta & Ray, 2003; Roberts, Montgomery, Lee, & Anderson, 2012). Cultural norms might exacerbate tobacco use when communities report benefits of tobacco use and lack models of its adverse impact on general and reproductive health (R. Gupta et al., 2013). *Jenu Kuruba* women from Mysore, Karnataka, for example, rely on smokeless tobacco for energy while working in agricultural fields and did not report any costs to reproduction (Placek et al., 2019).

*Jenu Kuruba* women report learning to chew tobacco for its perceived positive benefits and because prominent members of the community are tobacco users (Placek et al., 2019). Since cultural transmission via prestigious individuals can impact ingestive behaviours in pregnancy, this study draws upon the influence of members from a public health institute to transmit reproductive harms of tobacco to *Jenu Kuruba* women. This mode of transmission is predicted to be effective because individuals residing in rural Indian villagers associate biomedical knowledge with power and effectiveness (although this power can also instill caution; Nichter, 1980).

This study took place among Scheduled Tribe populations residing within Mysore District, Karnataka. Most were *Jenu Kurubas*, former hunter–gatherers who were displaced from their residential forest approximately 25–30 years ago (Roy, Hegde, Bhattacharya, Upadhya, & Kholkute, 2015). They were forced into government housing and were given plots of land to grow crops for either consumption or sale. They currently reside in small villages and are primarily employed as agricultural workers. Tribal populations in India, such as the *Jenu Kurubas*, are considered to be the most socially and economically disadvantaged members of society (Vijayalakshmi, 2003), and have been found to have higher levels of food insecurity, worse sanitation and less education than neighbouring rural farmer women (Placek et al., 2017).

Many members of the study population work in tobacco production. Approximately 77% of reproductive-aged women use some type of tobacco, rates that are equal to male *Jenu Kurubas*, and they are more likely to chew ‘natural’ tobacco products, such as loose leaf tobacco (*kaddipudi*), *paan* (tobacco, areca nut, slaked lime and betel leaves) and *hoggesoppu* (fresh tobacco) (Placek et al., 2019). Several participants were *Soligas*, who were formerly semi-nomadic and engaged in shifting cultivation (Pfeffer & Behera, 2015). Now, *Soligas* mainly harvest and sell non-timber forest products such as honey and bamboo. Finally, a few participants belonged to Scheduled Castes, who had married into *Jenu Kuruba* families.

Data were collected March through April 2016. *Jenu Kuruba* women frequently engage in agricultural work at distant locations. Women were therefore recruited using convenience sampling with the assistance of local public health educators and Accredited Social Health Activists (ASHA) workers. ASHA workers are trained by the National Rural Health Mission in India to liaise with the public health system, help launch public health programmes and educate women in their communities (Ministry of Health & Family Welfare-Government of India, 2020).

### Study design

The study had an experimental pre-test/post-test design with cluster randomization. ASHA workers identified reproductive-aged women in each *Jenu Kuruba* village (*haadi*), and arranged a day and time for all participants in a single village (a presentation group) to be interviewed separately (baseline) and then to gather together to view one of two presentations on tobacco harms (intervention). They then arranged a second day and time, about 10 days later, for all women to be interviewed again (followup). Participants were paid 150 rupees for completing the baseline interview and intervention and 100 rupees for completing the followup interview.

#### Baseline

Women completed a structured questionnaire that measured demographic indicators and use of tobacco and other substances, and knowledge of health risks from tobacco use. Participants also provided passive drool to assess cotinine, a nicotine metabolite that is a widely used biomarker of recent nicotine exposure, whose half-life is about 16 h (Benowitz, Hukkanen, & Jacob, 2009).

Because women’s food and substance use preferences during pregnancy and their reproductive years often vary according to factors such as resource scarcity, increased work demands, somatic constraints and cultural norms that encourage the consumption of certain substances over others (Patil, Abrams, Steinmetz, & Young, 2012; Placek et al., 2017, 2019), we assessed several of these factors at baseline to serve as control variables and to explore predictors of tobacco use vs. non-use. For the text of the questionnaire, see the Supplementary Information (SI).

#### Intervention

Immediately following the individual baseline interviews and biomarker collection, all participants in a presentation group were given one of two 10 min PowerPoint presentations on the harms of smokeless tobacco use. These harms were determined prior to the start of the study, based on a review of the literature on tobacco harms specific to India.

The general harms presentation (GHP) discussed mouth and throat cancer, tooth loss, stomach and esophagus cancer, heart disease and increased blood pressure. The reproductive harms presentation (RHP) discussed higher rates of preterm birth, lower birth weight, increased rates of stillbirths, lower fertility, early menopause, interference with fetal brain development and increased blood pressure during pregnancy. Both presentations had 13 slides and the same layout and colour scheme (see the SI). The slides were translated from English into *Kannada*, and then back-translated into English to confirm accuracy. To enhance content recollection, presentations were followed by a short group discussion asking participants to name five harms in the presentation. Each presentation was delivered by a trained counsellor from PHRII who had over a decade of experience providing reproductive health education to rural communities.

To minimize contamination between participants in the same village viewing different presentations and talking about them afterwards, all but two of the presentations were exclusive to a single village (the exceptions were on different days). Thus, our study was a cluster randomized trial, which involves randomizing at the group level rather than the individual level. The GHP and RHP were alternated sequentially between villages, so that approximately half of the participants viewed the GHP, and half the RHP. The slides of each presentation are provided in the SI.

#### Followup

About 10 days after the intervention, participants completed a structured questionnaire that assessed the use of tobacco, knowledge of harms of tobacco use and tobacco cognitions, i.e. how often they had tobacco cravings, and thought about cutting back or quitting, and the consequences of tobacco use. They were also asked if they had shared the presentation with anyone. Finally, participants provided a second saliva sample.

The number of days between baseline and followup varied somewhat between groups owing to unforeseen circumstances. Several pregnant participants, for example, were at the hospital for checkups or were going into labour, and therefore completed followup at a later date. For the full questionnaire, see the SI.

### Outcome measures

#### Tobacco use

At baseline and followup, we asked participants if they currently used any type of tobacco (yes/no), which types they used (freelist) and the number of times they had used any tobacco product in the last 24 h. We then collected saliva samples for cotinine assays (participants rinsed mouth with water, waited 10 min and provided saliva via passive drool).

Saliva samples were stored at −20°C on the day of collection at PHRII. Salivary cotinine concentrations were assayed using Salimetrics ELISA cotinine kits at the PHRII laboratory according to the manufacturer’s instructions. In brief, all standards and samples were assayed in duplicate. After analysis, five samples were removed from the dataset owing to apparent pipetting errors. Coefficients of variation (CV) were computed for each sample (intra-assay) and for controls on each plate (inter-assay). The mean intra-assay CV was 6.8%, below the manufacturer-recommended mean of 10%, and the inter-assays CVs were 14% for the high control and 14% for the low control, both below the manufacturer-recommended 15%.

Women were classified as tobacco users both by self-report and by salivary cotinine concentrations exceeding a 3 ng/ml cutoff for smokeless tobacco use that is based on representative US data (Agaku & King, 2014). See the SI for further discussion of this cutoff value.

Funding was available to purchase four cotinine kits, which could test a total of 152 saliva samples. Because we required baseline and followup cotinine values for each participant, we could assay values for 76 of the 79 participants who remained in the study at followup. For technical reasons, however, we could only assay baseline and followup samples from 70 participants.^1^ We therefore removed a random selection of nine participants from cotinine testing (but these nine remained in analyses not involving cotinine values). In addition, five samples had pipetting errors, leaving a total of 65 participants with cotinine values.

#### Tobacco harms

We assessed knowledge of the harms of tobacco use at baseline and followup by asking each participant to freelist as many harms as they could (Quinlan, 2005). We aggregated essentially identical harms, e.g. ‘still birth’ and ‘abortion’ were both coded ‘pregnancy loss’, and ‘damaged food pipe’ and ‘dental cancer’ were both coded as ‘mouth or throat cancer’. We then computed the *salience* of each of these specific harms for each participant (Quinlan, 2005; Smith, 1993). *Salience* takes into account both the order an item was mentioned (e.g. first or last), and the total number of items mentioned. Specifically:

salience=(N+1−rank)/N

where *N* is the number of harms mentioned by that participant, and *rank* is its rank order (e.g. if a participant mentions five harms, the harm mentioned first has *rank* = 1 and *salience* = 1 and the one mentioned last has *rank* = 5 and *salience* = 0.2). If a harm was not mentioned by a participant, that harm was assigned a salience of 0 for that participant. For participants who did not freelist any harm, we created a dummy ‘nothing’ harm that had a salience of 1. To compute the composite salience score, the saliences for each harm were averaged across all participants.

#### Tobacco cognitions

At followup only, we used a three-point scale to ask how often (never, sometimes, often) participants thought about craving tobacco, cutting back, quitting and the health consequences of tobacco use.

### Statistical analyses

We used linear and generalized linear mixed effects regression models to test the effect of the intervention (presentation type) on the followup outcome, controlling for the baseline level and pregnancy status. To account for the cluster-based design, we included presentation group as a random effect in mixed effects models. Gaussian and logistic models were fit using the lme4 package (Bates, Mächler, Bolker, & Walker, 2015), and Poisson and negative binomial models were fit using the glmmTMB package (Brooks et al., 2017).

We conducted exploratory analyses of predictors of tobacco use using elasticnet regression, a technique developed for analyses in which the number of predictors is large relative to the number of cases (Friedman, Hastie, & Tibshirani, 2010). For a brief description of this model, see the SI. We set the mixing parameter *α* = 0.2 and chose the regularization parameter *λ* by cross-validation.

All analyses were conducted in R version 3.6.3 (2020–02-29; code available from https://github.com/grasshoppermouse/mysore2016tobacco and data available from https://doi.org/10.5281/zenodo.3987496).

### Ethical approval

This study was approved by the Institutional Review Boards of Washington State University and the Public Health Research Institute of India.

## Results

We first report baseline sociodemographic variables, patterns of tobacco use, and knowledge of tobacco harms, and then report the effect of the intervention on these patterns at followup.

### Baseline sociodemographic variables

At baseline, 92 women from 10 villages participated in the study, with four to 13 women per village. The sample consisted of 31 pregnant women and 61 non-pregnant women, 75 of whom were married, with the remainder single, divorced or widowed. Among 86 ever married participants, 41 were, or had been, in arranged marriages. Most participants were *Jenu Kurubas* (*n* = 67), several were *Soligas* (*n* = 18), and a few belonged to Scheduled Castes (*n* = 4). Most participants worked in agriculture (*n* = 79); three worked in tobacco production; several were housewives (*n* = 9); a few worked in education (*n* = 3); and one was a businesswoman.

Of the 92 women in the study, 47 were assigned to the GHP condition, and 45 were assigned to the RHP condition. In two cases, researchers recruited two presentation groups from a single village on different days; hence, there was a total of 12 presentation groups. Followup occurred an average of 10 days after the presentation (range 7–15; SD = 2.2). At followup, there were 79 women (86% of the original sample) available for interviews, 41 in the GHP condition, 14 of whom were self-reported tobacco users, and 38 in the RHP condition, 15 of whom where self-reported tobacco users.

To check if attrition was biased, we tested for differences in the mean values of sociodemographic variables and baseline levels of key outcome variables in the participants at baseline, and those remaining at followup. Mean differences in all variables were small and not statistically significant, suggesting that attrition was not biased. See Table 1 for summary statistics.

### Baseline tobacco use patterns

About half of the non-pregnant women self-reported current use of tobacco (31 of 61) whereas about a sixth of pregnant women did (5 of 31). Of the 36 women who self-reported current tobacco use, 32 reported using *kaddipudi*, which is local tobacco that has been ground into a powder and is then chewed; one used *Madhu*, which is commercial tobacco powder, also chewed; and four used betel nut, which is a concoction of betel nut, betel leaf, slaked lime and often tobacco (one woman said she used betel nut without tobacco). After tobacco, the most commonly used substances were betel nut (*n* = 32) and alcohol (*n* = 6); all women who used alcohol also reported using tobacco.

Most participants reported that it is wrong for women to use tobacco (*n* = 87), but a few reported that it was acceptable (*n* = 4), and one woman did not know. As for the justification for why women should not use tobacco, most women did not know (*n* = 83), a few women mentioned disease (*n* = 4) and one woman mentioned that it was morally wrong. Of the few participants who reported that it was acceptable for women to use tobacco, one said that everyone is addicted so the elders feel like it is not a bad habit, one said that it prevented ‘heat’ (a disease state under the local humoral theory of disease), and one said that it was fun. A few women reported using tobacco for medicinal purposes, such as dental pain (*n* = 7) or as an antiseptic (*n* = 1), and a few also reported using tobacco for ritual purposes (*n* = 8). A number of women reported that their mothers used tobacco (*n* = 22) and/or that their mothers-in-law did (*n* = 21).

Of the 32 women who reported reasons to avoid quitting tobacco (most, but not all of whom were current users), 22 reported that quitting made them lose interest in work, 11 reported that quitting increased dental pain, 10 reported that quitting caused headache, fever or other health problems and nine reported that quitting caused poor mood (women could mention multiple reasons).

**Figure 1.**
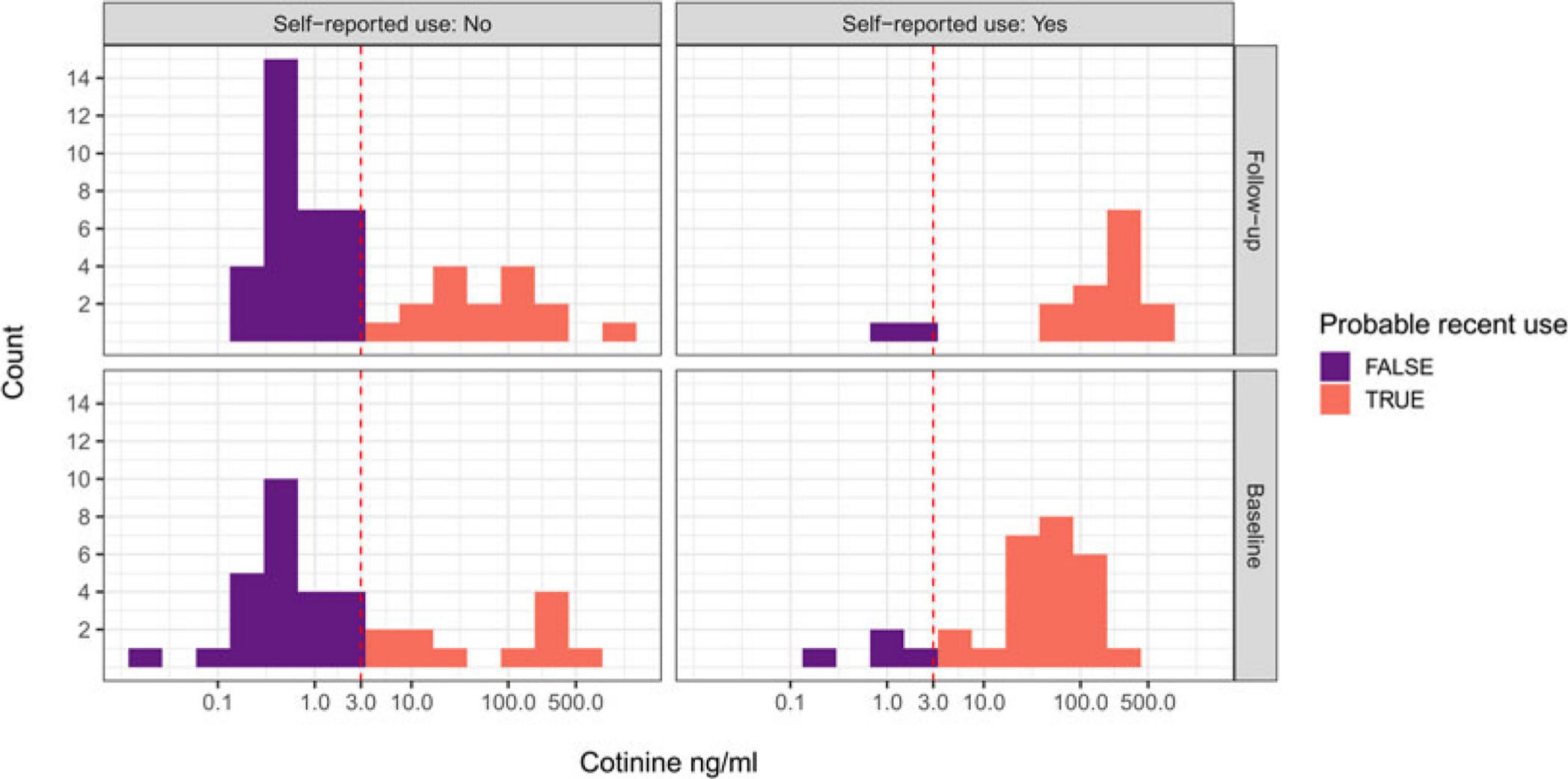
Histograms of baseline and *followup* salivary cotinine concentrations compared *with* self-report tobacco use in the last 24 h. Purple, Probable non-use of tobacco; red, probable use of tobacco. Based on 3 ng/ml cutoff (vertical dotted line) for smokeless tobacco use derived from US data (Agaku & King, 2014).

Based on salivary cotinine concentrations at baseline, and using a 3 ng/ml cutoff for recent tobacco use (based on smokeless tobacco use in a representative US sample; Agaku & King, 2014), 64% of non-pregnant women and 43% of pregnant women were recent tobacco users. Based on values below the cutoff at both baseline and followup, 28 of 65 participants (43.1%) were probable non-tobacco-users. We classified the remaining participants as probable users (see Figure 1).

Several women who did not self-report tobacco use had cotinine values consistent with recent tobacco use. We coded women as under-reporting tobacco use if they self-reported no tobacco use in the last 24 h and yet had cotinine values ≥3 ng/ml, and did so at either baseline or followup. According to these criteria, 35% of women under-reported their recent tobacco use (Figure 1, left panels). Conversely, 7.7% of women over-reported their recent tobacco use (Figure 1, right panels).

#### Exploratory analyses

We used a logistic elasticnet regression model to explore baseline tobacco user status as a function of age, pregnancy status, tobacco use by family or friends, education, income, marital status, arranged marriage, number of children, hours of domestic work, hours of non-domestic work and number of harms mentioned at baseline. Positive predictors of tobacco user status included older age, higher income, and a greater number of children, whereas being married or pregnant were negative predictors. For details, see the SI and Figure S4.

### Baseline knowledge and salience of tobacco harms

**Figure 2.**
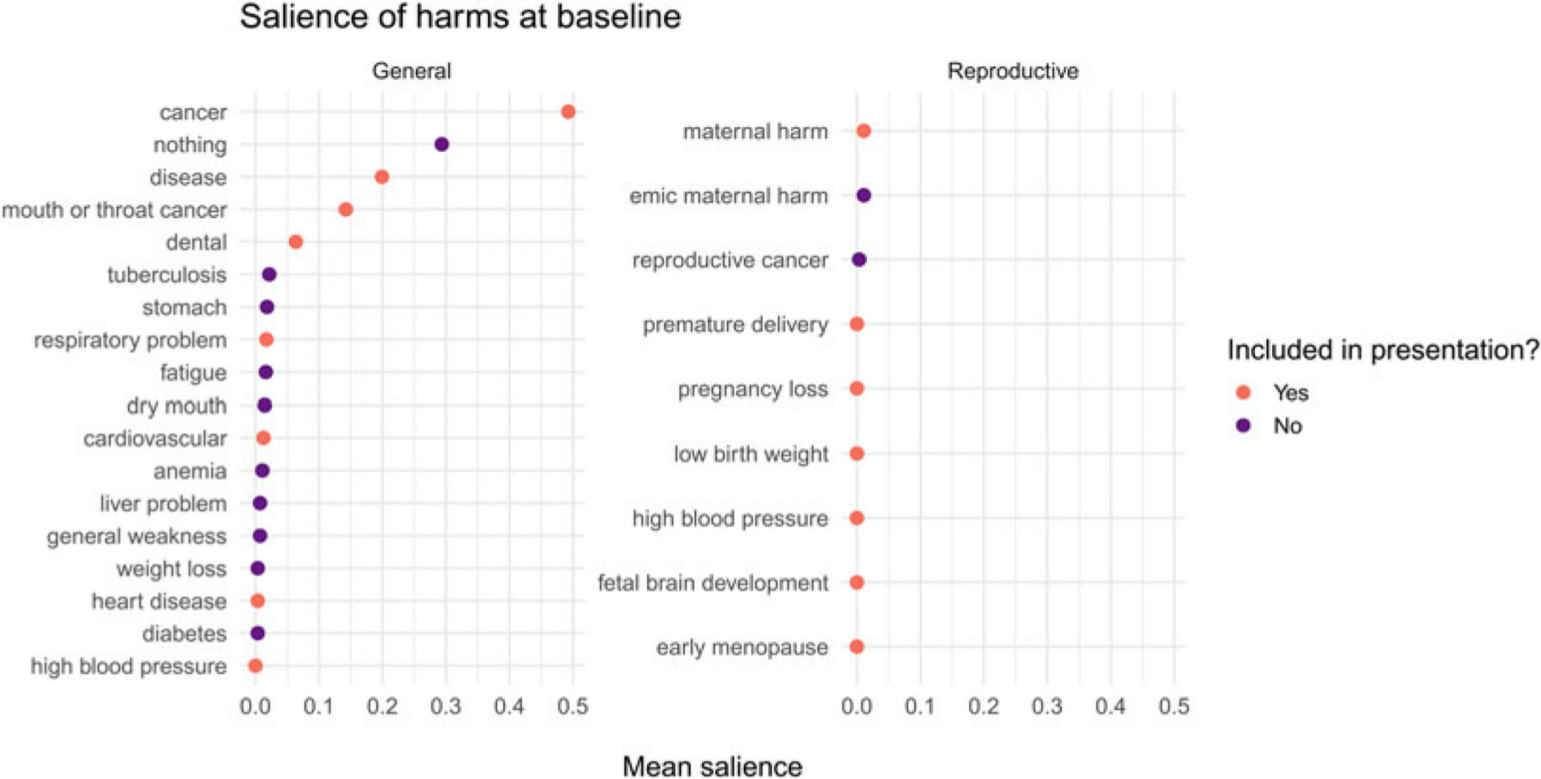
The salience of general and reproductive harms at baseline. Harms include both those free-listed by participants and those in the presentations that will be subsequently viewed. Dark symbols: Harms free-listed by participants at baseline that were not in either presentation. Many participants did not free-list any harm, which we coding as ‘nothing’, with a salience of 1. We arbitrarily included the mean salience of ‘nothing’ in the general harms panel.

We predicted that baseline knowledge of the reproductive harms of tobacco would be low. At baseline, 65 women (71%) free-listed at least one harm of tobacco use, and 27 women (29%) did not free-list any harm. The mean number of free-listed harms was mean (*M*) = 1.4 (standard deviation, SD = 1.1). After lightly aggregating harms (mentioned at either baseline or followup) that were essentially identical, participants freelisted 20 distinct harms (see Figure 2).

We computed the mean salience of harms that were freelisted at baseline before viewing the presentation. All harms in our GHP except high blood pressure were freelisted by at least one participant. Some general harms were familiar to many participants. Cancer, for example, was free-listed by 50 women. As predicted, very few participants freelisted reproductive harms, and if they did they were vague, e.g. ‘maternal harm’ (see Figure 2).

There was no significant difference in number of reported baseline harms by trimester or non-pregnant status (*χ*^2^ = 3.7, d.f. = 4, *p* = 0.45).

### Testing prediction 1: did the intervention reduce tobacco use, and more so in the reproductive than general condition?

At both baseline and followup, participants were asked how many times they had used tobacco in the last 24 h. Because this was an overdispersed count variable, we fit a negative binomial mixed effects regression model to test if presentation type had a significant effect on self-reported frequency at followup, controlling for baseline self-reported tobacco use and trimester-pregnant status, with a random intercept for presentation group. We expected that a reduction, if any, would be proportional to baseline use, i.e. heavier users would experience a larger reduction. We therefore included an interaction term. We found no significant effect of presentation type on self-reported tobacco use (see Table 2, Tobacco self-report model).

**Figure 3.**
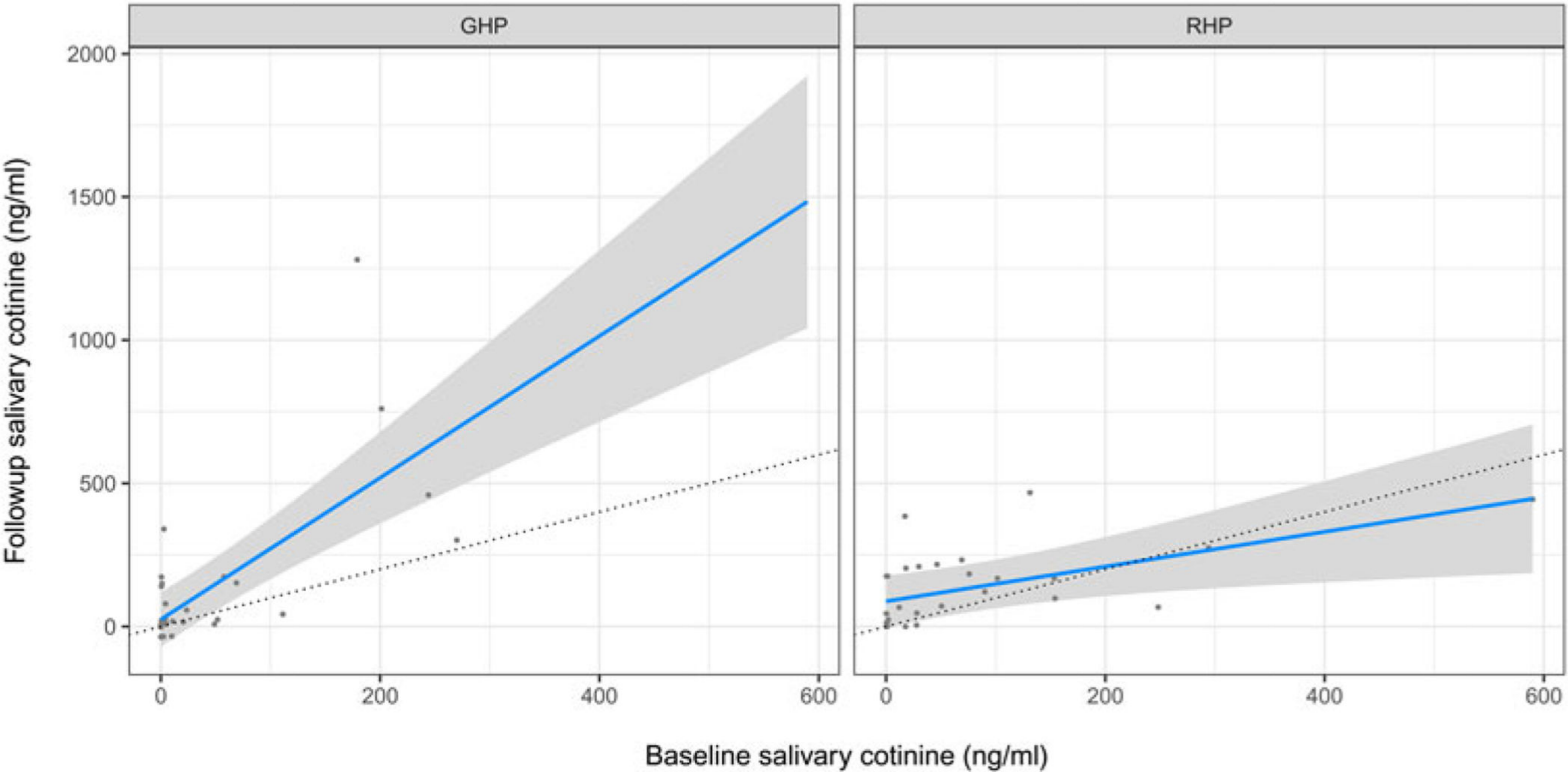
Linear mixed-effects regression model of followup cotinine as a function of baseline cotinine in the General (left panel) vs. Reproductive (right panel) health presentation conditions controlling for trimester. Dotted lines indicate followup cotinine equal to baseline. For regression coefficients, see Table 2, Cotinine 2 model.

Followup cotinine concentrations were significantly correlated with baseline concentrations (Pearson’s *r* = 0.51, Spearman’s rank correlation = 0.8; both *p*-values < 0.001). We tested if presentation type had a significant effect on cotinine concentration, controlling for baseline cotinine concentration and trimester. There was no significant main effect of presentation. We found a significant interaction, however, such that mean followup cotinine was higher than baseline cotinine in the GHP but was approximately equal to baseline in the RHP (see Figure 3 and Table 2, Cotinine 1 and Cotinine 2 models).

There was no significant main effect of trimester/non-pregnant status, but compared with the first trimester, followup cotinine levels were significantly higher among non-pregnant women (see Table 2, Cotinine 2 model).

Finally, because individuals who exhibited the greatest increases in number of free-listed harms at followup were arguably most heavily influenced by the presentations, we fit an exploratory model of followup cotinine as a function of the changes in number of general harms and reproductive harms mentioned at followup, relative to baseline. Increases in free-listed reproductive harms, but not general harms, were significantly negatively associated with followup cotinine (see Table S1 and Figure S5).

### Testing prediction 2: did the intervention increase knowledge of reproductive tobacco harms more than general harms?

Of the 79 participants remaining at followup, 73 (92.4%) free-listed at least one harm, and six (7.6%) did not free-list any harm. The mean number of freelisted harms was *M* = 2.5 (SD = 1.1), an average increase of about one harm over baseline, *M* = 1.4 (SD = 1.1), a statistically significant increase (*p* = 2.5 × 10^−7^).

Because our outcome was a count variable, and an overdispersion test did not reject the null hypothesis of equidispersion, we fit a mixed effects Poisson regression model of the number of harms freelisted at followup as a function of the presentation condition (GHP vs. RHP), the type of harm (general or reproductive), an interaction term for the presentation type and harm type, controlling for the number of harms freelisted at baseline and pregnancy status/trimester, and a random intercept for presentation group.

**Figure 4.**
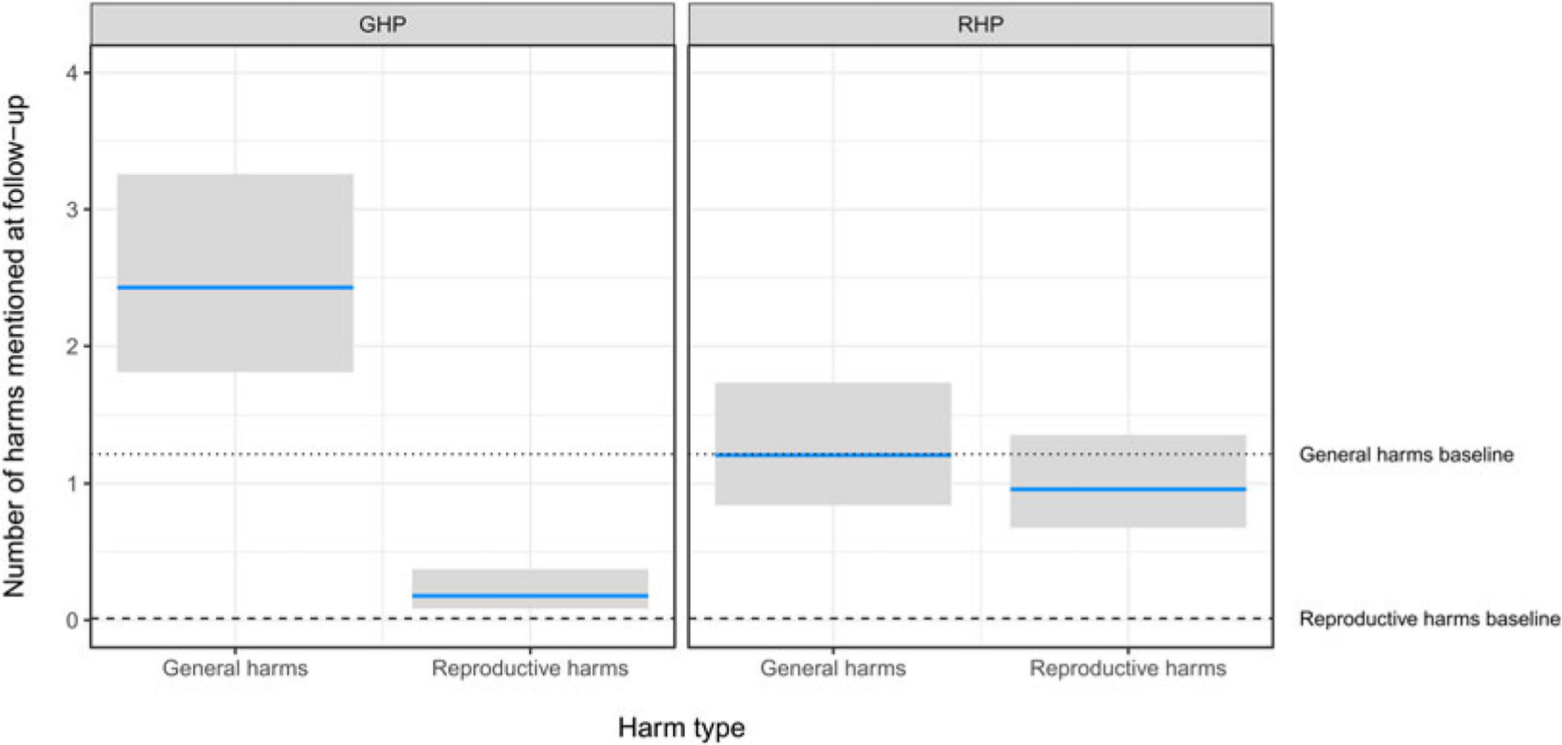
Poisson mixed-effects regression of number of general and reproductive harms mentioned at followup, by type of presentation, controlling for number of such harms mentioned at baseline and trimester, and with a random intercept for presentation group. Grey bars represent the 95% CI. GHP: General harms presentation. RHP: reproductive harms presentation. See Table 2, *Number of harms* model.

As predicted, the GHP condition improved knowledge of general harms and the RHP condition improved knowledge of reproductive harms. Participants in the GHP condition mentioned about two general harms at followup, which represented an increase in knowledge of about one general harm over baseline, and they mentioned less than one reproductive harm, close to their baseline performance. Participants in the RHP condition mentioned about one reproductive harm at followup, which represented an increase of about one reproductive harm over their baseline performance, and about one general harm, which was close to their baseline performance. Contrary to predictions, the RHP condition did not enhance knowledge more than the GHP condition. There was no significant main effect of trimester or non-pregnant status (see Figure 4 and Table 2, *Number of harms* model).

We explored if pregnancy status or trimester was associated with the number and/or type of harms mentioned at followup. We found no significant associations (tests not reported).

### Exploratory analysis of change in salience of specific reproductive and general harms at followup compared with baseline

For each specific harm free-listed by participants, we computed the difference in mean salience at followup compared with baseline (Δ*salience*). We computed 95% confidence intervals for Δ*salience* using 1000 bootstrap replications.

**Figure 5.**
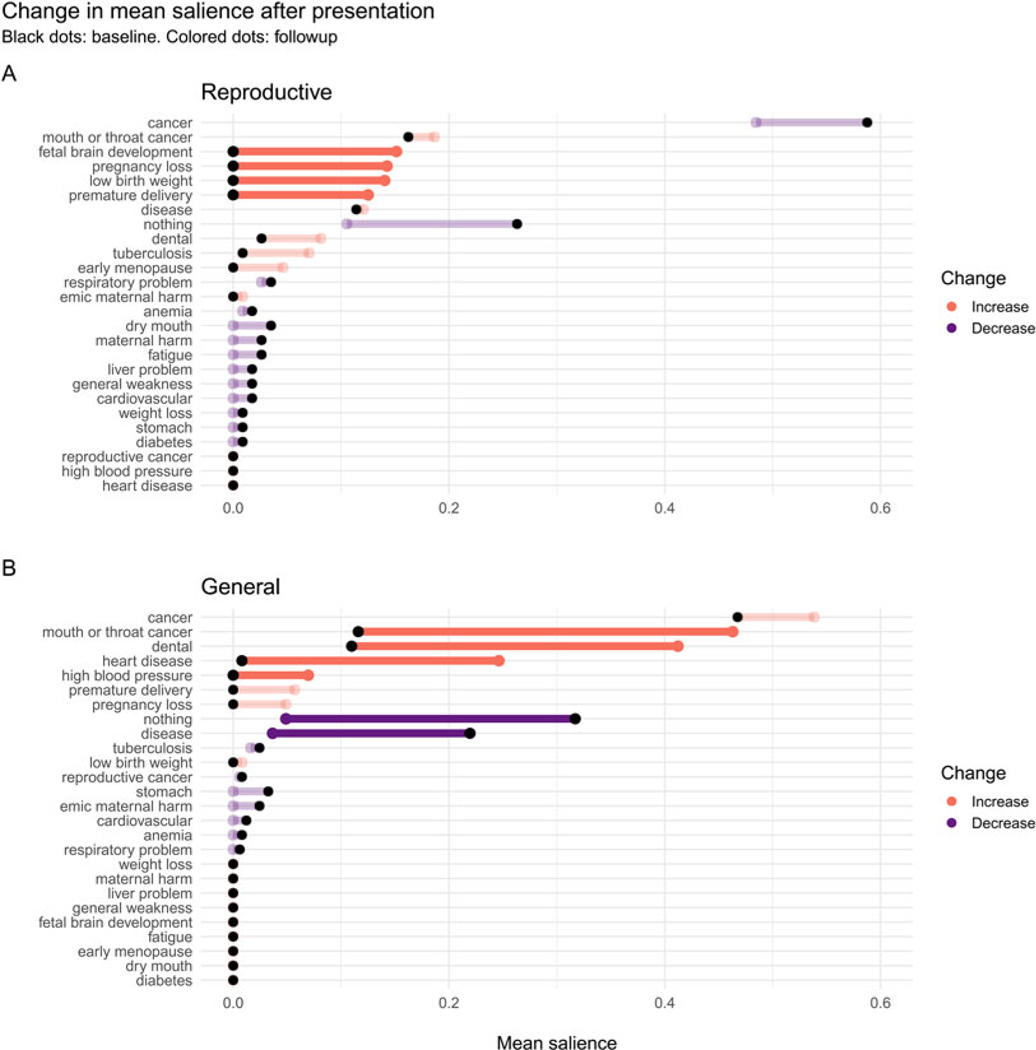
Mean salience of each harm at followup (*coloured* dots), relative to baseline mean salience (black dots), in the Reproductive (top) and General (bottom) health presentation conditions. Harms not mentioned at baseline or followup have a salience of 0 for that time point. *The* 95% CI of Δ*salience* was estimated by bootstrapping. Faded lines: Change in salience was not significant (95% CI of Δ*salience* included 0).

For participants in the RHP condition, the mean salience of cancer and other general harms decreased, as did the salience of ‘nothing’ (although their saliences remained greater than zero), whereas the salience of many specific reproductive harms, such as pregnancy loss, fetal brain development and low birth weight, increased from zero. For participants in the GHP condition, the salience of cancers increased from their positive values at baseline to even higher values; the salience of heart disease and high blood pressure increased from their near-zero baseline levels; and the salience of one reproductive harm, pregnancy loss, also increased from its zero baseline level, indicating that some participants in the RHP condition probably spoke with participants in the GHP condition prior to the followup interview. The salience of ‘nothing’ and generic ‘disease’ decreased sharply. Hence, each presentation was effective at increasing knowledge and salience of specific tobacco harms and decreased the number of participants who failed to free-list any harm (see Figure 5).

### Testing prediction 3: did the intervention alter tobacco cravings and intentions to quit, and more so in the reproductive than general conditions?

**Figure 6.**
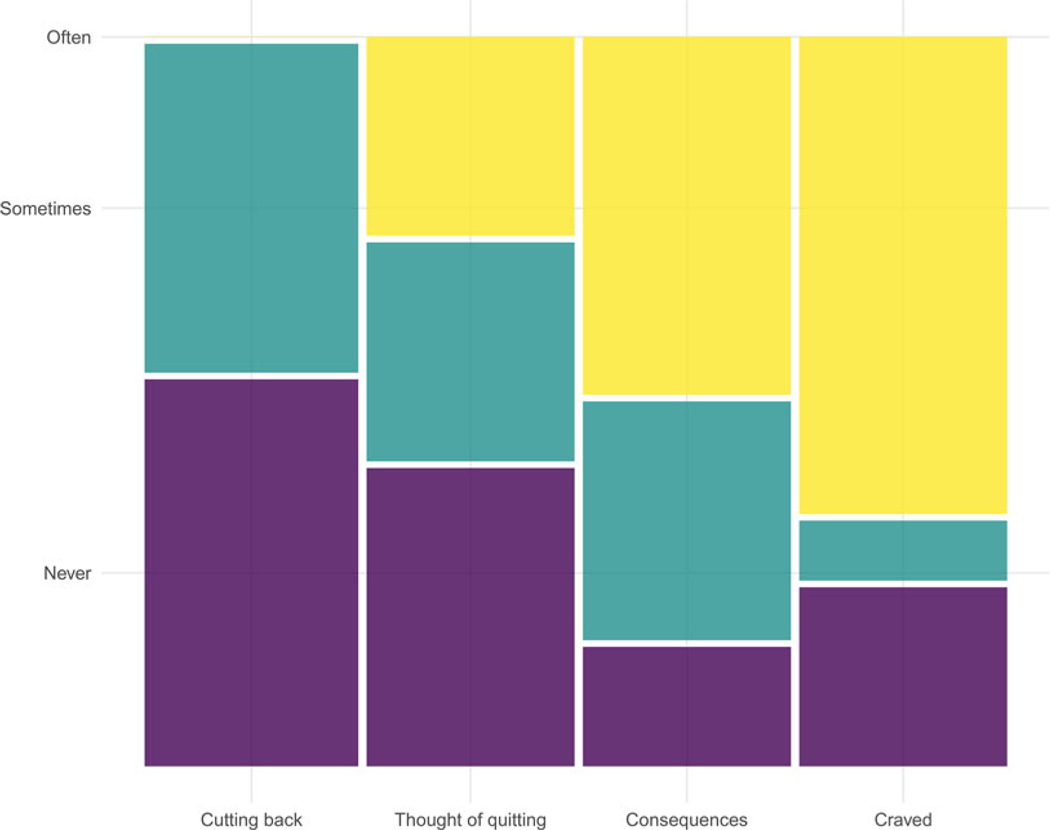
The proportions of recent tobacco users that often, sometimes, or never thought about cutting use, quitting, or the consequences of tobacco use.

After removing probable non-users from the followup sample, the proportions of the remaining 37 participants that indicated they craved tobacco and thought about the health consequences of tobacco use, cutting their use and quitting, are depicted in Figure 6. We found no evidence that participants in the RHP condition were more (or less) likely to report intentions to reduce or eliminate tobacco use than those in the GHP condition (all *p*-values > 0.05; tests not reported).

### Testing prediction 4: were participants in the reproductive condition more likely to share the presentation than those in the general condition?

Of the 79 participants at followup, 65 reported that they shared information from the presentation with someone else, e.g. a relative, friend or neighbour. We fit a mixed effects logistic regression model of sharing information as a function of the number of harms mentioned at followup, presentation condition (RHP or GHP) and trimester. Participants in the RHP condition were slightly but not significantly more likely than those in GHP condition to share information from the presentation. The more harms a participant free-listed at followup the more likely she was to share the information with others. There was no significant effect of trimester or non-pregnant status (see Table 2, *Share presentation* model, and Figure S6).

## Discussion

In one of the few cotinine-verified studies of tobacco use among tribal women in India, we found that an alarming 64% of non-pregnant women and 43% of pregnant women were recent tobacco users. Moreover, a substantial 35% of women under-reported their recent tobacco use at either baseline or followup (Figure 1, left panels), a phenomenon that has been documented in several populations (Gorber, Schofield-Hurwitz, Hardt, Levasseur, & Tremblay, 2009). We investigated if an anti-tobacco messaging strategy that emphasized near-term reproductive harms would be more effective than a standard messaging strategy in reducing tobacco use, increasing knowledge of harms, increasing intentions to quit and promoting sharing of information about harms. Our results were mixed, but generally indicated that, although most women were not aware of reproductive harms, and that both short presentations were effective at increasing knowledge of harms, the RHP was not more effective than the GHP on the outcomes of interest.

Our prediction that the RHP would reduce tobacco use compared with the GHP was supported (see Table 2, Cotinine 2 model), but this result was mainly driven by a few women in the GHP condition who had high cotinine levels at baseline and even higher levels at followup, and a few women in the RHP condition who had high baseline levels but lower levels at followup (see Figure 3). Thus, this finding does not constitute strong evidence for prediction 1.

This finding also had some odd patterns. We expected a coefficient of baseline cotinine on followup cotinine of about 1 in the GHP group, but instead it was about 2 (see Table 2, *Cotinine* 2 model). This was a longitudinal study in a rural indigenous population that produces tobacco. Population access to tobacco could conceivably have changed over the course of the 10 days between baseline and followup, e.g. owing to payment of wages or crop harvesting. If the GHP group took advantage of increased access to tobacco, but the RHP did not (because the latter group was now aware of the reproductive harms), this could explain the increase in the former group but no increase in the latter group. Another possibility is that women knew they would attend a presentation on tobacco and they might have abstained at baseline. However, at followup there was no presentation, only a short interview, so perhaps the GHP group did not abstain a second time but the RHP group did (again, because the latter group was now aware of the reproductive harms). Because cotinine values are so variable, though, it is likely that this increase is simply noise. Unfortunately, we do not have the data to investigate the increase in cotinine concentrations in the GHP group. Finally, we found no evidence that participants in the RHP condition were more (or less) likely to reduce or eliminate self-reported tobacco use than those in the GHP condition.

Consistent with our expectations, most women failed to mention any reproductive harms of tobacco use at baseline. Instead, cancer was by far the most salient harm, followed by ‘nothing’ (see Figure 2). About 10 days after viewing a brief presentation, however, 92.4% of women freelisted at least one harm, and on average freelisted 2.5 harms, which represented an increase of approximately one harm over baseline. As predicted, the GHP improved knowledge of general harms, and the RHP improved knowledge of reproductive harms, as evidenced by the number and type of harms free-listed from baseline to followup (see Figure 4 and Table 2, *Number of harms* model).

However, although the brief RHP was effective at increasing awareness of the reproductive harms of tobacco use, it was not *more* effective than the GHP at increasing the total number of harms mentioned at followup, contrary to predictions (see Figure 4 and Table 2, *Number of harms* model). Nevertheless, despite the fact that cancer remained by far the most salient harm even in the RHP group, reproductive harms had become quite salient. Consistent with the fetal protection hypothesis, the most salient reproductive harms were to fetal brain development, pregnancy loss, low birth weight and premature delivery, whereas early menopause and reproductive cancers were less salient (see Figure 5a).

There are at least three possible reasons why tobacco harms related to oral health were particularly salient at baseline, and increased in the GHP condition (see Figure 5b). First, there is an epidemic of oral cancer in India, and this is salient in tribal regions throughout India (B. Gupta et al., 2013; Mohan & Lando, 2016), particularly among women and in tribal regions (Deepa et al., 2013; Deo et al., 2017; P. C. Gupta et al., 1998; Mukhopadhyay, 2006). Second, dental appearance and oral health, e.g. tooth whiteness and gingivitis, are significant components of physical attractiveness (Joiner & Luo, 2017; Montero et al., 2014), which is important in many social domains, including romantic relationships (Buss & Schmitt, 2019; Eastwick, Luchies, Finkel, & Hunt, 2014). The negative impact of tobacco use on physical attractiveness, in turn, is an important motivation to quit tobacco use (Magnan, 2017, and references therein). Third, pregnant women are at increased risk of developing oral health problems, such as gingivitis, dental caries and periodontal disease (Silk, Douglass, Douglass, & Silk, 2008). These risks are heightened owing to fluctuations in estrogen and progesterone, increased acidity in the dental cavity owing to vomiting, and increased cravings for and consumption of sugar (Gambhir, Nirola, Gupta, Sekhon, & Anand, 2015; Silk et al., 2008). Oral health problems in pregnancy are associated with adverse birth outcomes because women can pass cariogenic bacteria to fetuses, and treating women for periodontal disease can lower rates of preterm birth (George et al., 2011). Currently, though, there is no evidence that women are are aware of these associations, which is a topic for future research. Like fetal protection, oral health therefore has near-term consequences for reproductive-age women in a population with high prevalence of oral health problems, both in its impact on physical attractiveness and in its implications for pregnant women.

Community-based research has found that peer-to-peer transmission of tobacco harms is an effective strategy in reducing use because peers are less intimidating than physicians (Secker-Walker et al., 2000; Williams et al., 2011). Most participants reported that they shared information from the presentations with others, although women in the RHP condition were not more likely to share than women in the GHP condition, contrary to predictions (see Figure S6 and Table 2, *Share presentation* model). This finding nonetheless indicates that sharing in this community is an effective strategy for communicating public health knowledge, and future research should consider using peer-to-peer transmission of tobacco harms to promote cessation in this community.

One exploratory result was particularly noteworthy. The women who were arguably most heavily influenced by the presentations where those who exhibited the greatest increases in the number of free-listed harms at followup relative to baseline. In support, an exploratory analysis found that an increase in the freelisted number of reproductive harms, but not general harms, was a significant negative predictor of cotinine concentrations at followup. See Table S1 and Figure S5.

A second exploratory analysis found that probable use of tobacco was positively associated with older age, income and number of children, and a few other variables, and negatively associated with being married and pregnant (see Figure S4). The positive effect of age is consistent with the higher prevalence of tobacco use seen globally in older women (Ng et al., 2014), and this pattern is consistent with the fetal protection hypothesis because the older women in our study were approaching the end of their reproductive careers (and therefore would be less likely to be, or become, pregnant; Hagen et al., 2013, 2016). The positive effect of income with use is consistent with gender inequality models (Hitchman & Fong, 2011), but the positive effect of number of children is not. The negative effect of married status with tobacco use is also seen in other populations (Ramsey Jr, Chen-Sankey, Reese-Smith, & Choi, 2019), and the negative effect of pregnancy status is consistent with the fetal protection model.

Although the RHP was effective at increasing knowledge, it generally did not outperform the GHP. There are several possible explanations. First, contrary to our hypothesis, reproductive harms might not be more salient to reproductive-aged women than general harms, especially harms like cancer that are deadly. Second, because most women were aware of at least one general harm of tobacco use but none were aware of any specific reproductive harm, the RHP was providing new knowledge whereas the GHP was often repeating existing knowledge, and repetition is known to increase judgments of truth (Dechêne, Stahl, Hansen, & Wänke, 2010). A greater perceived truth of general harms might have outweighed the putatively greater salience of reproductive harms. Third, in retrospect, some of the most salient general harms, such as mouth cancer and dental problems, reduce facial attractiveness. Attractiveness has near-term consequences for mating and other relationships, and the negative effects of tobacco use on attractiveness motivate quitting (Magnan, 2017). Further, as noted earlier, some general harms are associated with perinatal complications. Thus, the general harms might have overlapped with reproductive harms to a certain extent, reducing the differences between the presentations.

### Limitations

The study had a relatively small sample size. Although we endeavoured to restrict each village to a single presentation, two villages had two presentations, and there was some evidence that information leaked from the RHP to the GHP condition: at followup, a few women in the GHP condition mentioned reproductive harms that they did not mention at baseline. This could have reduced differences between the RHP and GHP conditions, and hence our ability to detect an effect of the intervention. Some women’s self-reports of tobacco use were unreliable, probably owing to cultural norms that limit reporting. Although our measure of cotinine indicated under-reporting, we remain uncertain of women’s frequency of tobacco use because we only collected two samples about 10 days apart. Furthermore, our exploratory analyses were unable to detect factors contributing to under-reporting by individual participants. Nevertheless, almost all participants said that it was wrong for women to use tobacco. Given that the research assistants who conducted interviews were connected to a public health institution, it is therefore likely that participants were worried about the stigma attached to tobacco use, or perhaps cautious about the power associated with biomedical knowledge (Nichter, 1980), and this motivated some to under-report their use. Furthermore, participants probably use more than one system of medical knowledge (e.g. humoral theory), perhaps ones in which tobacco is viewed as beneficial, and these other systems could have influenced their responses or their continued use of tobacco. Finally, although women retained knowledge of the previously unknown reproductive harms for 10 days, similar studies have found that the effects of interventions aimed at changing tobacco use behaviours do not last long (Bull et al., 2014).

## Conclusion

The prevalence of tobacco use among women in LMICs is currently low, and this demographic is therefore increasingly targeted by tobacco companies. It is imperative to develop public health initiatives to counter tobacco advertising and maintain low rates of tobacco use. Unfortunately, health behaviour change interventions are often ineffective in low-income groups (Bull et al., 2014). Evolutionary theories predict that individuals in adverse conditions more steeply discount the future, and that the substance use decisions of reproductive-age women are heavily influenced by information about reproductive harms. We therefore used a cluster-randomized controlled design to test if a presentation on tobacco harms that emphasized near-term reproductive harms, such as pregnancy loss, would have a greater negative impact on subsequent tobacco use than one that emphasized general harms that tend to occur later in life, such as cancer.

As predicted, we found that a disadvantaged population of reproductive-age women in South India with a high prevalence of tobacco use were mostly unaware of tobacco’s reproductive harms, and that information from a brief presentation on such harms was salient and retained 10 days later. Contrary to predictions, we found that presentations on reproductive and general harms had approximately equal effects on subsequent increased knowledge of harms of tobacco use. Nevertheless, an intriguing exploratory analysis found that women who listed an increased number of reproductive harms at followup had significantly lower cotinine levels. Future studies of tobacco control messages should take into account the psychological impact of the messages and the local disease ecology, such as the high rate of oral cancer in the study population, and should incorporate harms that have near-term consequences for young people, such as negative impacts on attractiveness, and for women, negative impacts on pregnancy.

## Supplementary Material

1

2

3

4

5

## Figures and Tables

**Table 1.** Summary statistics for sample demographics and key outcome variables for original sample and those remaining at followup (*d*, Cohen’s *d* for mean difference; *p*, *p*-value for mean difference)

	Baseline			Followup			Difference	
			
Variable	*N*	Range	Mean (SD)	*N*	Range	Mean (SD)	*d*	*p*
Age (years)	92	18–40	29 (7.6)	79	18–40	29 (7.5)	−0.0056	0.95

Education (years)	91	0–15	4.9 (4)	79	0–15	5 (4)	−0.0210	0.92

Monthly income (1000 rupees)	91	2–30	9.5 (5.2)	79	2–30	9.3 (5.2)	0.0430	0.69

Months pregnant	31	2–9	5.7 (2.3)	25	2–9	5.6 (2.4)	0.0250	0.95

Number of children	92	0–5	1.9 (1.4)	79	0–5	1.8 (1.3)	0.0270	0.88

Daily hours of domestic work	91	0–10	3.3 (2.1)	79	0–10	3.4 (2.2)	−0.0300	0.88

Daily hours of non-domestic work	92	0–10	5.1 (3.2)	79	0–10	5 (3.2)	0.0410	0.83

Tobacco use age of onset	39	10–37	19 (5.8)	31	10–37	20 (6.3)	−0.0570	0.97

Number of tobacco use harms (baseline)	92	0–4	1.4 (1.1)	79	0–4	1.4 (1.1)	−0.0270	0.87

Self-reported frequency of recent tobacco use (baseline)	92	0–30	5.4 (8.7)	79	0–30	4.8 (8.1)	0.0790	0.69

**Table 2.** Mixed effects regression models of the effect of the intervention on different outcomes, as specified in the table headings. Values are estimated coefficients (standard errors). All models included presentation type (the intervention), control for pregnancy status and a random intercept for presentation group

	Tobacco self-report	Cotinine 1	Cotinine 2	Number of harms	Share presentation
(Intercept)	−1.46 (1.03)	−24.84 (74.28)	−146.33* (71.73)	0.78** (0.27)	−0.13 (1.51)
Trimester 2	−0.12 (1.41)	22.22 (94.97)	129.97 (85.42)	0.12 (0.34)	−0.18 (1.71)
Trimester 3	1.12 (1.11)	77.73 (100.41)	177.07* (89.29)	−0.21 (0.36)	−1.75 (1.59)
Not pregnant	1.23 (1.05)	98.91 (77.21)	175.44* (70.75)	0.11 (0.27)	−1.11 (1.44)
RHP	−0.32 (0.52)	−15.59 (51.50)	63.14 (50.02)	−0.70*** (0.18)	1.04 (0.94)
Baseline tobacco frequency	0.07* (0.03)				
Baseline tobacco frequency × RHP	0.04 (0.03)				
Baseline cotinine		1.13*** (0.24)	2.47*** (0.39)		
Baseline cotinine × RHP			−1.87*** (0.47)		
Baseline number of harms				0.03 (0.08)	
Number of reproductive harms				−2.62*** (0.40)	
Number of reproductive harms × RHP				2.38*** (0.45)	
Total number of harms at followup					1.10** (0.41)
Number of observations	79	65	65	158	79

* *p* < 0.05

** *p* < 0.01

*** *p* < 0.001.

*Base levels*: Trimester 1 and General harms presentation (GHP). *Tobacco self-report*: Negative binomial mixed-effects regression model of self-reported tobacco use frequency at followup, controlling for self-reported baseline frequency. *Cotinine 1*: Linear mixed-effects regression model of followup cotinine concentrations, controlling for baseline cotinine concentrations. *Cotinine 2*: Same as Cotinine 1, except includes an interaction term between baseline cotinine and presentation condition (see Figure 3). *Number of harms*: Poisson mixed effects regression model of the total number of harms mentioned at followup, controlling for number of harms mentioned at baseline. Harms distinguished by general vs. reproductive. This model has double the number of observations because there were two separate counts of harm per participant (reproductive and general); the random effect was thus participant nested within presentation group (see Figure 4). *Share presentation*: Logistic regression model of sharing information from the presentation as a function of presentation type and the number of harms mentioned at followup. Coefficients are log odds (see Figure S6). For more model statistics, see Table S2. RHP: Reproductive harms presentation.

## Footnotes

1 In each kit, a set of standards was assayed along with the samples. Technicians used the first kit to assay two sets of samples at different times, which thus involved assaying two sets of standards, thereby reducing the number of samples that could be assayed
