# Supplementary material for: The impact of information about tobacco-related reproductive vs. general health risks on South Indian women's tobacco use decisions
Source: Evol Hum Sci. 2020 Nov 20;3:e4. doi: 10.1017/ehs.2020.61 (PMC7996064; doi:10.1017/ehs.2020.61)
Supplement: Supplementary file 1 [file S2513843X20000614sup001.zip › Follow-up questionnaire_tobacco study.docx]

1. Name:
2. In the last week, how often have you thought about quitting your tobacco use?
   1. Never
   2. Sometimes
   3. Often
3. How many times have you used tobacco today?
4. Have you successfully quit using any type of tobacco? If so, please explain.
   1. Yes
   2. No
5. In the last week, how often have you thought about the consequences of using tobacco?
   1. Never
   2. Sometimes
   3. Often
6. In the last week, how often have you craved tobacco?
   1. Never
   2. Sometimes
   3. Often
7. In the last week, has anyone encouraged you to use tobacco? If yes, who?
8. In the last week, has anyone discouraged you from using tobacco? If yes, who?
9. In the last week, how often have you thought about cutting back on your tobacco use?
   1. Never
   2. Sometimes
   3. Often
10. What methods have you used to cut back and/or quit tobacco use?
11. Have you told anyone about what you learned in the presentation about tobacco use?
12. Please tell me what content you remember from the presentation.
