# Supplementary material for: The impact of information about tobacco-related reproductive vs. general health risks on South Indian women's tobacco use decisions
Source: Evol Hum Sci. 2020 Nov 20;3:e4. doi: 10.1017/ehs.2020.61 (PMC7996064; doi:10.1017/ehs.2020.61)
Supplement: Supplementary file 1 [file S2513843X20000614sup001.zip › supplementary information.pdf]

Supplementary information

Cotinine cutoff

The cotinine concentration distributions of users and non-users overlap. Non-users of tobacco can have non-zero concentrations of salivary cotinine due to exposure to environmental tobacco smoke (ETS), or, in a tobacco-producing region such as this, from handling tobacco leaves without protection. Tobacco users, on the other hand, can have low concentrations of salivary cotinine if they have not recently used tobacco (the half-life of cotinine is about 18 hours). Because some individuals misreport their tobacco use, it can be challenging to distinguish users from non-users based on cotinine concentrations alone. A wide range of cutoffs that attempt to optimize sensitivity vs. specificity have been reported in different studies and different ethnic groups, in part because levels of ETS have changed over time (generally decreasing due to tobacco control efforts), and cotinine concentrations in non-users have therefore also changed over time (Kim, 2016). To our knowledge, there is no study of the optimal cotinine cutoff value in a large, representative sample of the Indian population. We therefore estimated an optimal value from our data and compared it to the 3 ng/ml value reported in a large, representative study of US oral tobacco users (N = 30,298; Agaku & King, 2014).

In our study, cotinine concentrations were bimodally distributed (see Figure S1). We therefore fit a Gaussian mixture model to log 10 cotinine values using the mixtools package (Benaglia, Chauveau, Hunter, & Young, 2009). Gaussian mixture models assume that each observation is generated by one of K mixture components with probability π_k, where each component is a Gaussian distribution, $N(\mu_k, \sigma_k)$. The parameters $\{\mu_k, \sigma_k, \pi_k\}$ are estimated from the data using the iterative expectation-maximization (EM) algorithm (for details, see Benaglia et al., 2009). In our data, using the default settings, the `normalmixEM` function determined that there were $K = 2$ components, which we interpreted as tobacco non-users (left) vs. users (right). See Figure S1.

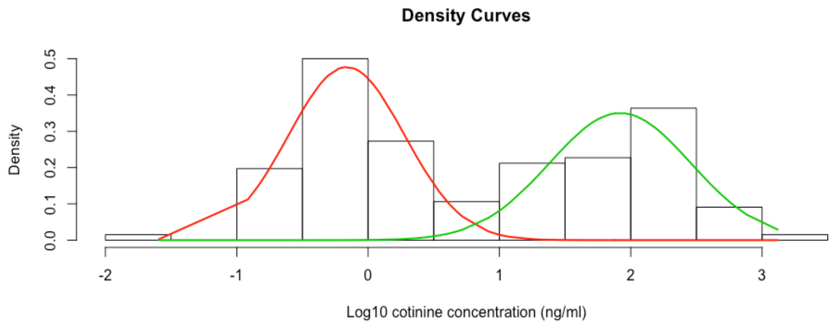

Figure S1: Histogram of log 10 cotinine concentrations overlaid with the two Gaussian components (red, green) estimated from the data using the `normalmixEM` function from mixtools.

We plotted the probability that each observation was generated by each component. The cotinine value for which there was an equal probability of belonging to the left vs. right component was 6.5 ng/ml (dotted line, Figure S2), i.e., women with cotinine concentrations less than this were more likely to belong to the non-user component, whereas those with concentrations greater than this were more likely to belong to the user component.

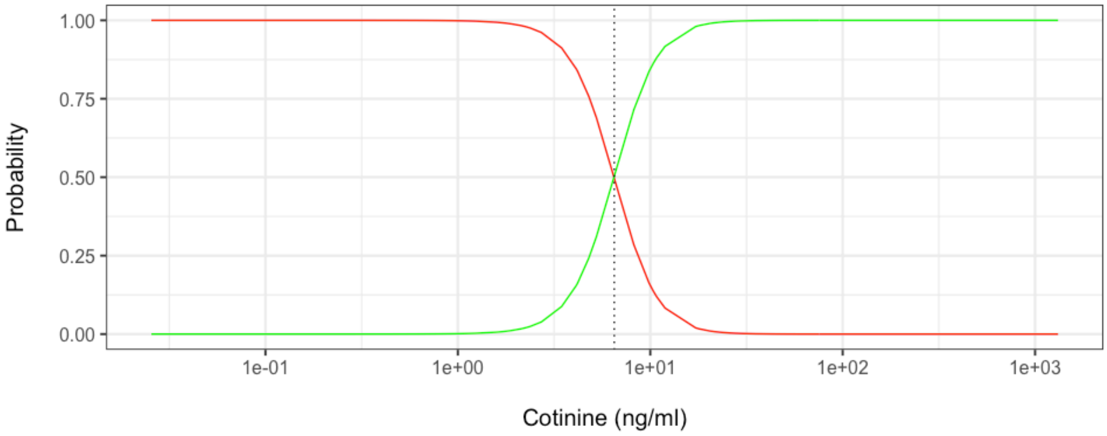

Figure S2: Probabilities of belonging to the non-user (left) vs. user (right) component based on cotinine concentration. The dotted line represents the cotinine concentration for which there was an equal probability of belonging to either component.

The 6.5 ng/ml value is close to the optimal 3 ng/ml cutoff estimated from the large US study. Although the 6.5 ng/ml value has the advantage of being estimated directly from the population in this study, it has the disadvantage of being based on a small, non-representative sample. Using the 6.5 ng/ml value also did not substantially change the percent of women who under-reported their tobacco use, 31%, vs. 35% for the 3 ng/ml value. We therefore chose to use the 3 ng/ml cutoff to distinguish probable non-users of tobacco from probable users.

Post-hoc power analysis

At the request of the editor we conducted a post-hoc power analysis of the effect of the intervention on cotinine concentrations in the treatment vs. control groups. We did not conduct a power analysis prior to the study because we had very little information on the proportion of female tobacco users in this population, the distributions of cotinine concentrations, or the extent to which women were aware of the harms of tobacco use.

Using information acquired in this study, we estimated power curves using simulations as follows. In our sample, half the women were non-users and half were users. We used the parameters of the two Gaussian distributions of log cotinine among non-users and users, respectively, as estimated by the mixture model (Figure S1), to simulate baseline cotinine values for equal numbers of non-users and users. We then randomly assigned equal numbers of simulated participants to the RHP treatment and GHP control conditions. We computed followup cotinine as a linear function of baseline cotinine, condition, their

interaction, and a Gaussian error term (residual error), similar to our actual linear regression analysis (except without a *Trimester* term).

$$cotinine_{followup} = \beta_0 + \beta_1 cotinine_{baseline} + \beta_2 RHP + \beta_3 cotinine_{baseline} RHP + \mathcal{N}(0, \sigma)$$

We computed power using three sets of parameters for the linear function. First, we used the parameters estimated from our real (non-simulated) data (very similar to those reported in Table 2, Cotinine 2 model, except without a *Trimester* term). Important disadvantages of these parameters are that (1) they are noisy estimates of the true parameters, e.g., of the effect of the presentation on the slope (the interaction term), and hence there will be substantial uncertainty in the estimate of power, and (2) the estimated negative effect of the presentation on the slope was very likely biased toward a more negative value due to selection based on statistical significance. In addition, these parameters were surprising from a theoretical perspective. We predicted that the coefficient of baseline cotinine, β_1, would be about 1. Instead, it was about 2, i.e., the cotinine concentrations of participants in the GHP condition were about double their baseline levels. The effect of the RHP condition was to reduce this effect by about 75% (the coefficient of the interaction term, β_3, was about -1.5). The power to detect the (likely biased) effect of the RHP condition, i.e., the p-value for β_3, with our sample size of 66, and $\alpha = 0.05$ was very high, 95%. See Figure S3.

The second set of parameters more closely matched our theoretical expectations, with a coefficient for baseline cotinine, $\beta_1 = 1$, an important negative effect of the RHP of $\beta_3 = -0.75$, and residual variation as seen in our data ($\sigma = 169$). For our sample size, the power was modest, 63%.

The third set of parameters reflected a small effect: a coefficient of baseline cotinine, $\beta_1 = 1$, an interaction term, $\beta_3 = -0.5$, and residual variation as seen in our data ($\sigma = 169$). For our sample size, the power was low, 42%.

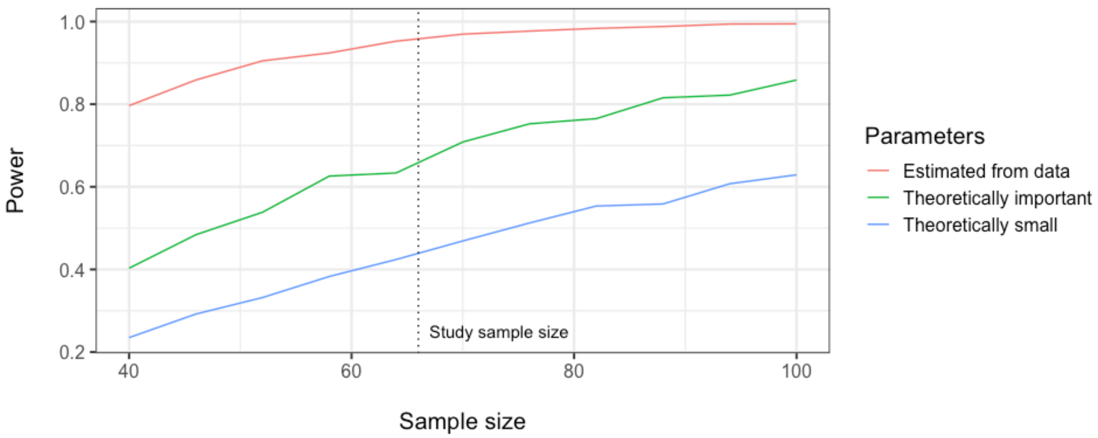

Figure S3: Power versus sample size for three sets of parameters and $\alpha = 0.05$ *(see text).*

We conclude that, given the actual prevalence of tobacco use among women in this population and their distribution of cotinine concentrations, our study was highly powered

to detect the effect we found, modestly powered to detect a theoretically motivated substantial effect, and poorly powered to detect a theoretically motivated small effect.

Exploratory elasticnet model of sociodemographic predictors of tobacco use

As described in the main text, we defined probable nontobacco users as participants who had cotinine values $< 3ng/ml$ at both baseline and followup. The remaining participants were classified as probable users. To explore sociodemographic predictors of tobacco user status, we fit an elasticnet logistic regression model with mixing parameter $\alpha = 0.2$ and λ chosen by cross-validation (Friedman et al., 2010).

We briefly describe the elasticnet regression model. Standard regression models are fit by minimizing an objective function. In ordinary least squares regression, the objective function is the residual sum of squares (RSS), and in logistic regression it is the negative log-likelihood, $-loglik(\beta)$. Penalized regression models instead minimize the objective function plus a penalty term based on the magnitude of the coefficient vector (Le Cessie & Van Houwelingen, 1992). For linear regression this is

$$\frac{1}{2} RSS/n + \lambda * penalty$$

and for logistic regression:

$$-loglik(\beta)/n + \lambda * penalty$$

There are two popular forms of penalized regression: ridge regression and lasso regression. For ridge regression the penalty is $||\beta||_2^2 = \sum_{j=1}^{p} \beta_j^2$, where the β_j are the regression coefficients, and for lasso regression the penalty is $||\beta||_1 = \sum_{j=1}^{p} |\beta_j|$. When $\lambda = 0$, this reduces to the standard estimation. As $\lambda \to \infty$, the coefficients β_j are "shrunk" to 0. Thus, when λ is small, the βs are relatively unrestricted, which can result in a good fit to the current sample (low bias), but a poor fit on future samples (high variance); roughly, the model will tend to be over-fitted. When λ is large, the βs tend to shrink toward 0, which reduces fit on the current sample (high bias) but results in a more stable fit across samples (low variance); roughly, the model will tend to be under-fitted. The optimal value of λ is typically found by minimizing cross-validation error.

With the lasso penalty, some coefficients might be set to 0, i.e., dropped from the model, which aids interpretation, but when variables are correlated, the lasso might drop some that are genuinely related to the outcome. In ridge regression, in contrast, the coefficients of correlated variables are shrunk to similar values; although the coefficients of some predictors might be very small, all predictors are retained in the model, which can make interpretation difficult.

Elastic net regression combines the advantages of ridge and lasso penalties using an additional tuning parameter α, $0 \leq \alpha \leq 1$:

$$penalty = (1 - \alpha)||\beta||_2^2/2 + \alpha||\beta||_1.$$

Thus, $\alpha = 0$ is the ridge penalty and $\alpha = 1$ is the lasso penalty. With intermediate values of α, there is a 'grouping' effect in which strongly correlated variables tend to enter or leave the model together (i.e., have their coefficients set to 0).

We used elastic net regression to fit a logistic regression model of tobacco use status as functions of our sociodemographic variables. Following standard procedure, we used 10-fold cross-validation to find the optimum value of λ, i.e., the one that minimized cross-validation error. We standardized our continuous variables (age, income, education years, number of children, domestic and non-domestic hours worked, and number of harms freelisted at baseline) by two standard deviations to approximate the standard deviations of our binary variables (married, pregnant, arranged marriage, and tobacco use by mother, mother-in-law, family, and friends), which improves comparisons of the coefficients of all variables (Gelman, 2008). See Figure S4.

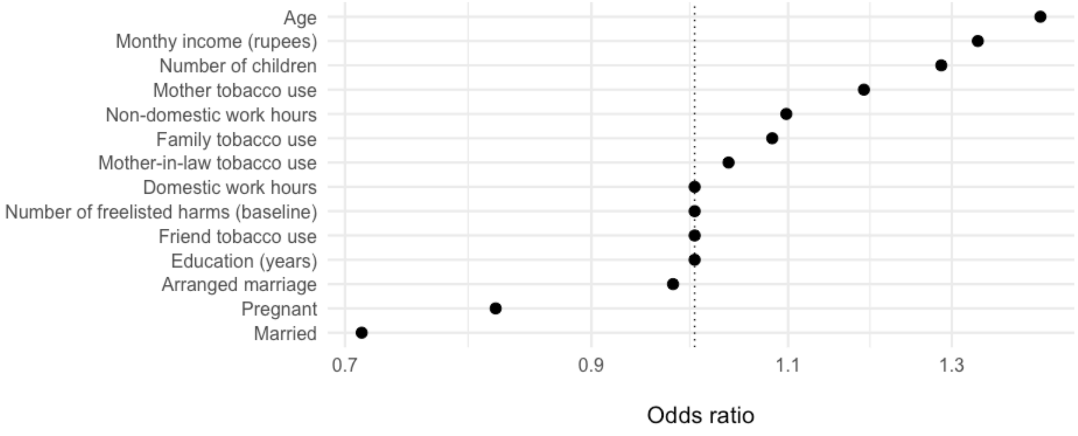

Figure S4: Logistic elasticnet regression coefficients of tobacco user status, fit using glmnet with $\alpha = 0.2$ and λ chosen by cross-validation. Continuous variables were centered and scaled by two standard deviations (see text). N=65

Exploratory model of followup cotinine as a function of changes in numbers of free-listed harms from baseline to followup.

Individuals who exhibited the greatest increases in number of free-listed harms at followup were arguably most heavily influenced by the presentations. We therefore fit an exploratory mixed effects linear regression model of log10 followup cotinine as a function of the changes in numbers of free-listed general harms and reproductive harms, controlling for log10 baseline cotinine and Presentation. Increases in the numbers of free-listed reproductive harms, but not general harms, were negatively associated with followup cotinine. See Table S1.

	Log10 Cotinine
(Intercept)	-0.03 (0.25)
Trimester 2	0.20 (0.31)
Trimester 3	0.19 (0.33)
Not pregnant	0.18 (0.25)
RHP	0.40* (0.18)
Δ Number of reproductive harms	-0.29** (0.10)
Δ Number of general harms	0.02 (0.06)
Num.Obs.	65

* p < 0.05, ** p < 0.01, *** p < 0.001

Table S1: Linear mixed effects model of log10 followup cotinine concentration as a function of the changes in numbers of general and reproductive harms free-listed at followup compared to baseline, controlling for log10 baseline cotinine and presentation condition. Values are estimated coefficients (standard errors).

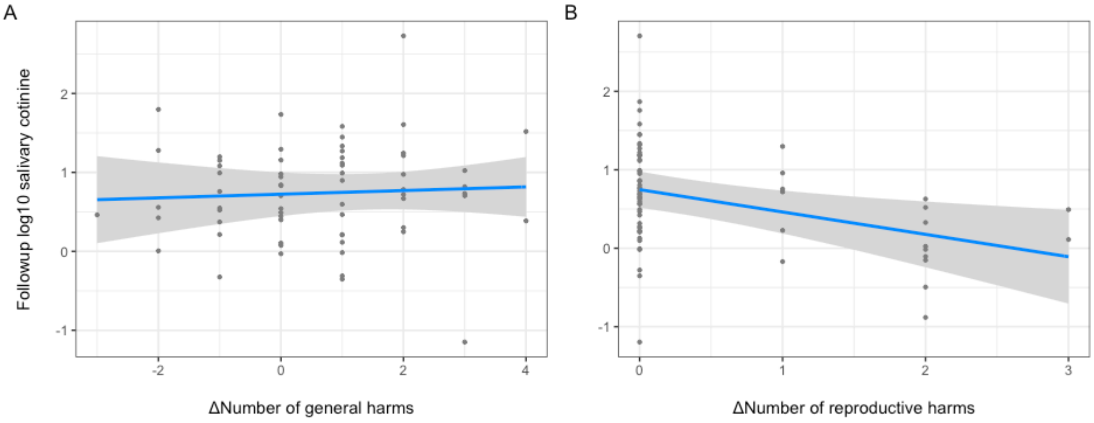

Figure S5: Effects plot for a regression model of log10 cotinine concentration versus the change in number of (A) general harms and (B) reproductive harms mentioned at followup compared to baseline (see Table S1).

Logistic regression model of sharing presentation

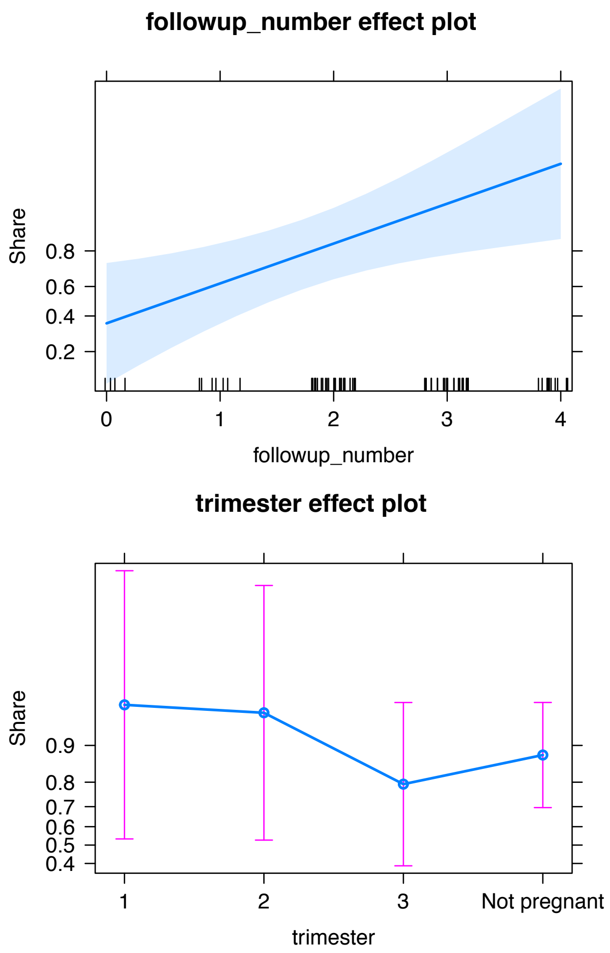

Figure S6: Logistic regression model of the effect of presentation type and the number of harms free-listed at followup on self-reported sharing of presentation with others. Base level is 'Did not share'. For regression coefficients, see Table 2, model Share presentation.

Expanded table of regression models

This table displays the coefficients of the same regression models as Table 2, but adds z-values, full p-values, and 95% CI's.

Variable	Estimate	SE	Z-value	P-value	Lower 95% CI	Upper 95% CI
Tobacco self-report						
(Intercept)	-1.46	1.03	-1.42	0.16	-3.49	0.562
Trimester 2	-0.12	1.41	-0.09	0.93	-2.89	2.65
Trimester 3	1.12	1.11	1.01	0.31	-1.05	3.3
Not pregnant	1.23	1.05	1.18	0.24	-0.822	3.29
RHP	-0.32	0.52	-0.63	0.53	-1.33	0.687
Baseline Tobacco Frequency	0.07	0.03	2.23	0.026	0.00808	0.125
Baseline Tobacco Frequency * RHP	0.04	0.03	1.18	0.24	-0.0265	0.107
Cotinine 1						
(Intercept)	-24.84	74.28	-0.33	0.74	-170	121
Trimester 2	22.22	94.97	0.23	0.82	-164	208
Trimester 3	77.73	100.41	0.77	0.44	-119	275
Not pregnant	98.91	77.21	1.28	0.21	-52.4	250
RHP	-15.59	51.5	-0.3	0.77	-117	85.3
Baseline cotinine	1.13	0.24	4.79	1.2e-05	0.67	1.6
Cotinine 2						
(Intercept)	-146.33	71.73	-2.04	0.041	-287	-5.74
RHP	63.14	50.02	1.26	0.21	-34.9	161
Trimester 2	129.97	85.42	1.52	0.13	-37.5	297
Trimester 3	177.07	89.29	1.98	0.047	2.06	352
Not pregnant	175.44	70.75	2.48	0.013	36.8	314
Baseline cotinine	2.47	0.39	6.27	3.6e-10	1.7	3.25
Baseline cotinine * RHP	-1.87	0.47	-3.96	7.6e-05	-2.79	-0.941
Number of harms						
(Intercept)	0.78	0.27	2.91	0.0036	0.254	1.3
Trimester 2	0.12	0.34	0.35	0.72	-0.543	0.782
Trimester 3	-0.21	0.36	-0.59	0.56	-0.908	0.49
Not pregnant	0.11	0.27	0.41	0.68	-0.413	0.631
RHP	-0.7	0.18	-3.88	1e-04	-1.05	-0.346
Baseline number of harms	0.03	0.08	0.42	0.67	-0.127	0.197
Number of reproductive harms	-2.62	0.4	-6.47	1e-10	-3.41	-1.82
Number of reproductive harms * RHP	2.38	0.45	5.29	1.2e-07	1.5	3.27
Share presentation						
(Intercept)	-0.13	1.51	-0.09	0.93	-3.1	2.83
RHP	1.04	0.94	1.1	0.27	-0.81	2.89
Trimester 2	-0.18	1.71	-0.1	0.92	-3.52	3.17
Trimester 3	-1.75	1.59	-1.1	0.27	-4.86	1.36
Not pregnant	-1.11	1.44	-0.77	0.44	-3.93	1.72
Total number of harms at followup	1.1	0.41	2.69	0.0071	0.298	1.9

Table S2. Regression coefficients, standard errors, z-values, p-values, and 95% confidence intervals for the models described in the main text and displayed in Table 2.
