# Supplementary material for: The impact of information about tobacco-related reproductive vs. general health risks on South Indian women's tobacco use decisions
Source: Evol Hum Sci. 2020 Nov 20;3:e4. doi: 10.1017/ehs.2020.61 (PMC7996064; doi:10.1017/ehs.2020.61)
Supplement: Supplementary file 1 [file S2513843X20000614sup001.zip › tobacco effects on reproductive health.pptx]

## Slide 1
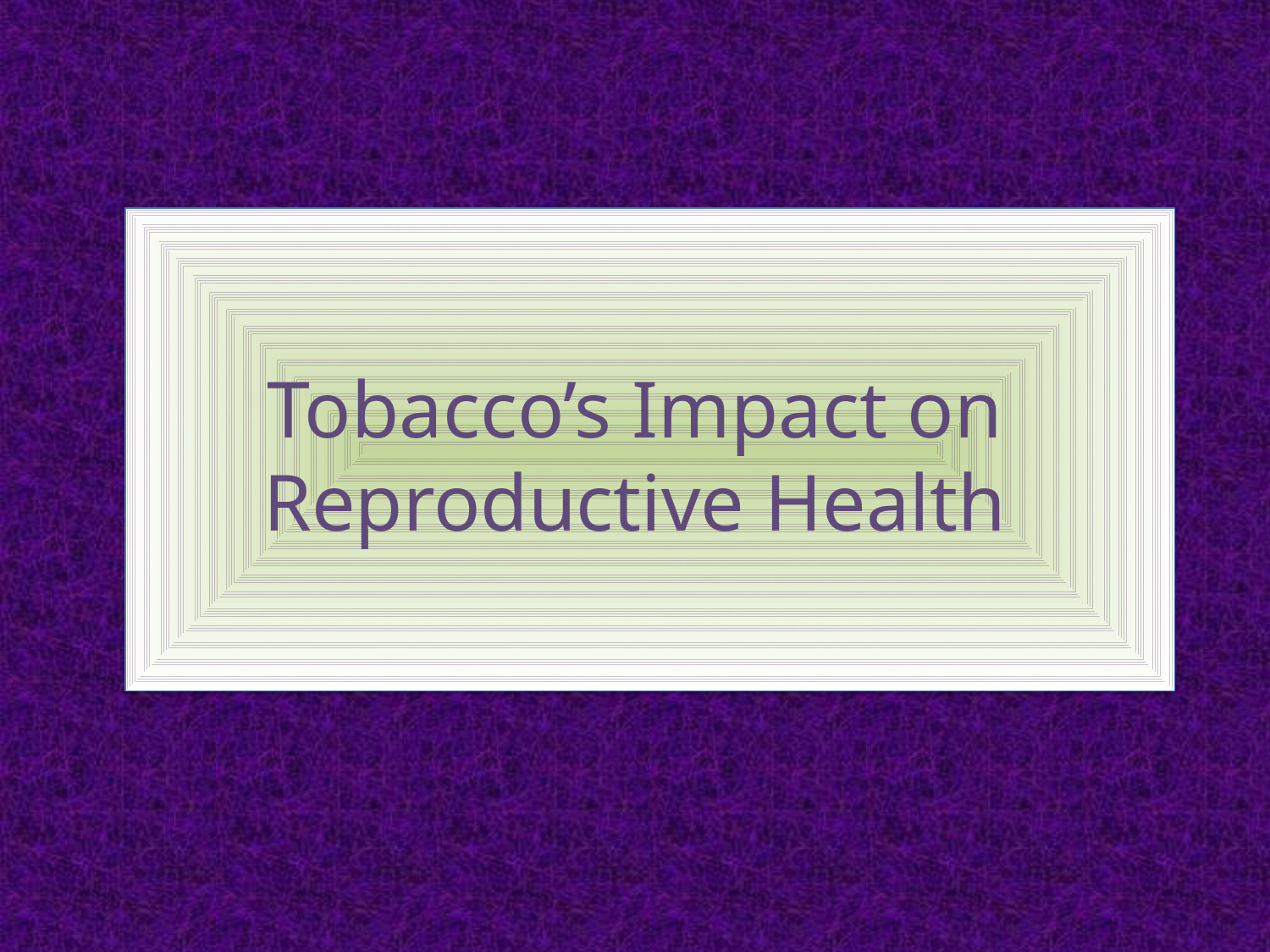

# Tobacco’s Impact on Reproductive Health

## Slide 2
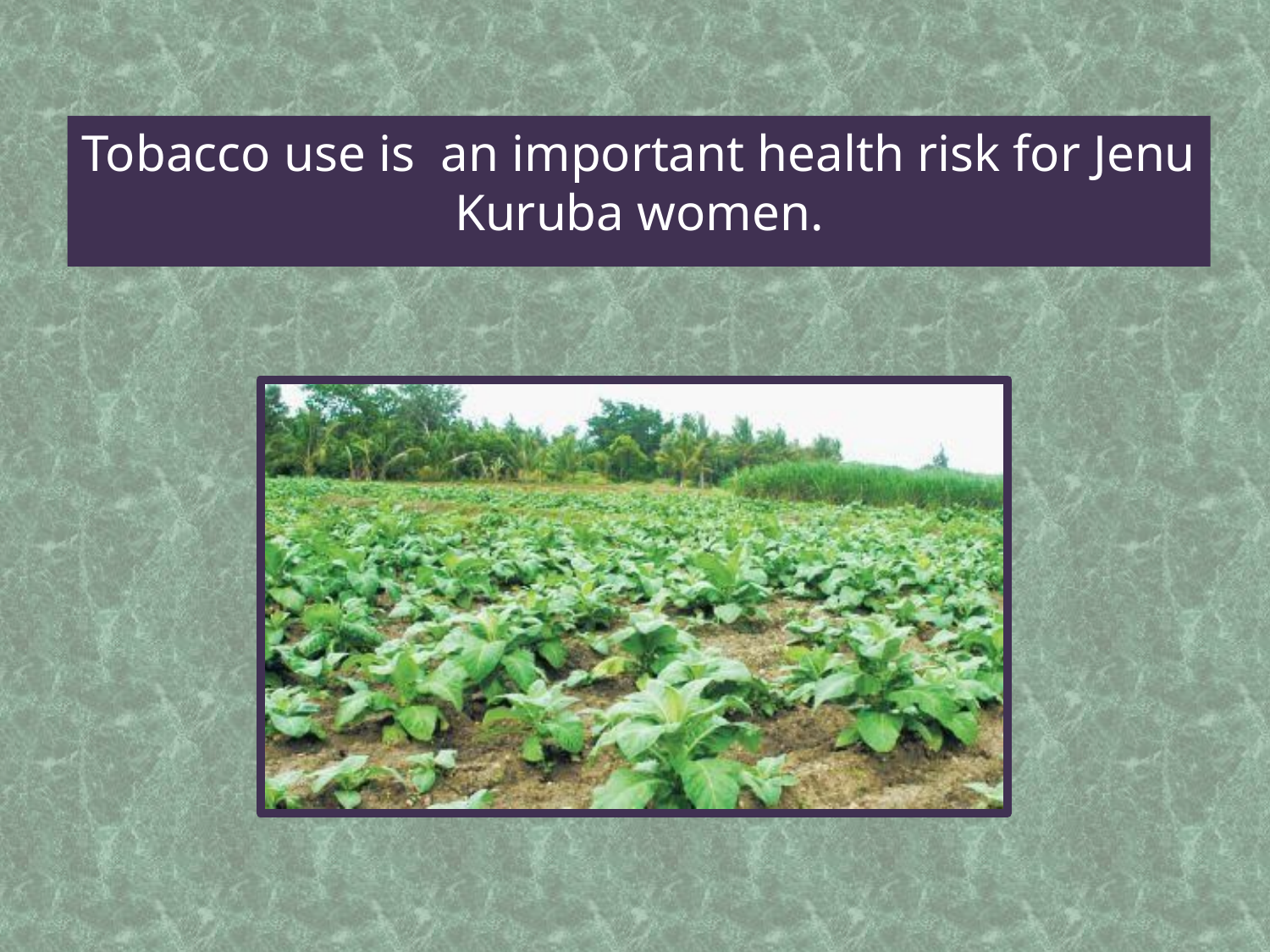

Tobacco use is an important health risk for Jenu Kuruba women.

## Slide 3
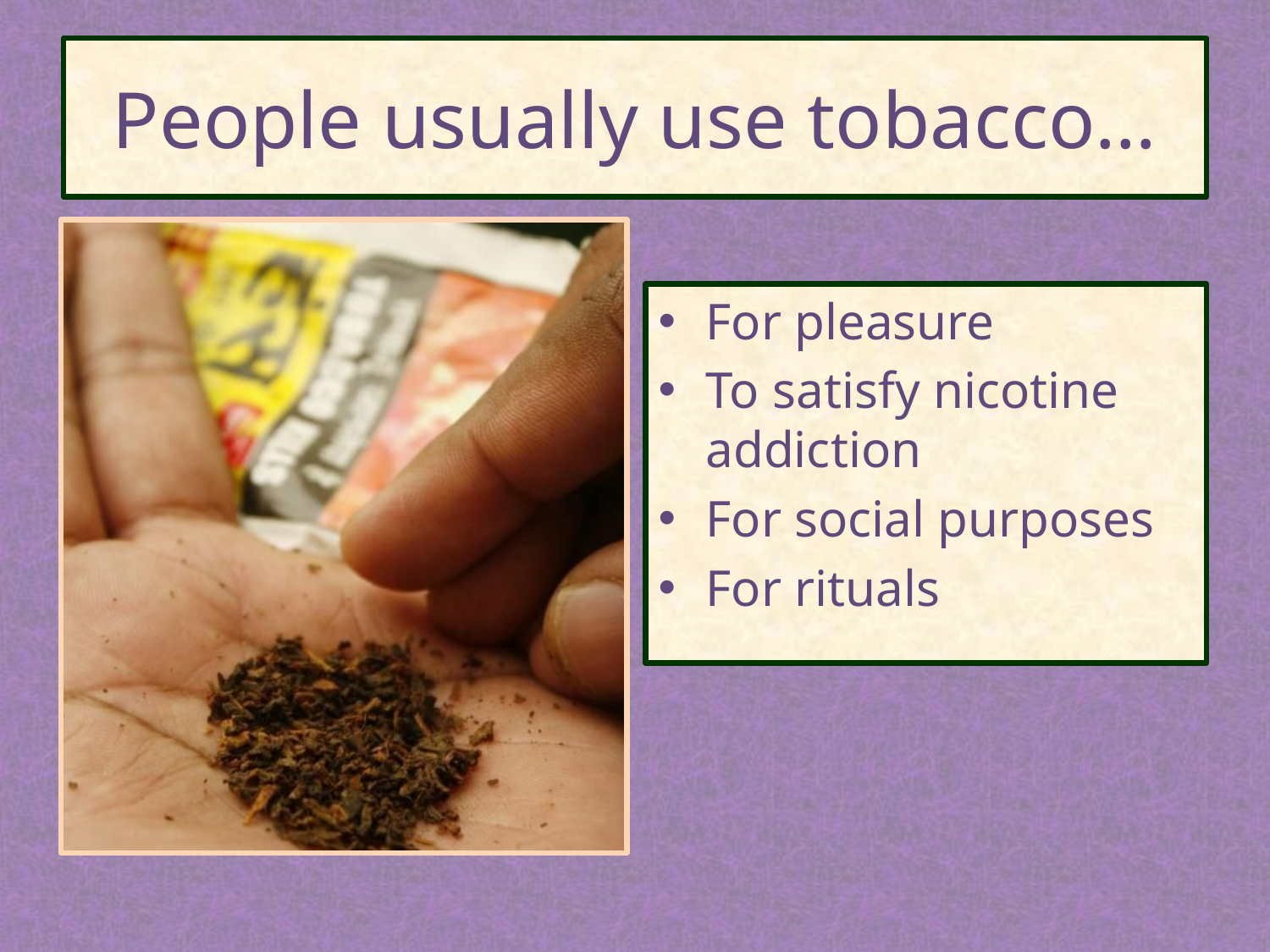

# People usually use tobacco…
For pleasure
To satisfy nicotine addiction
For social purposes
For rituals

## Slide 4
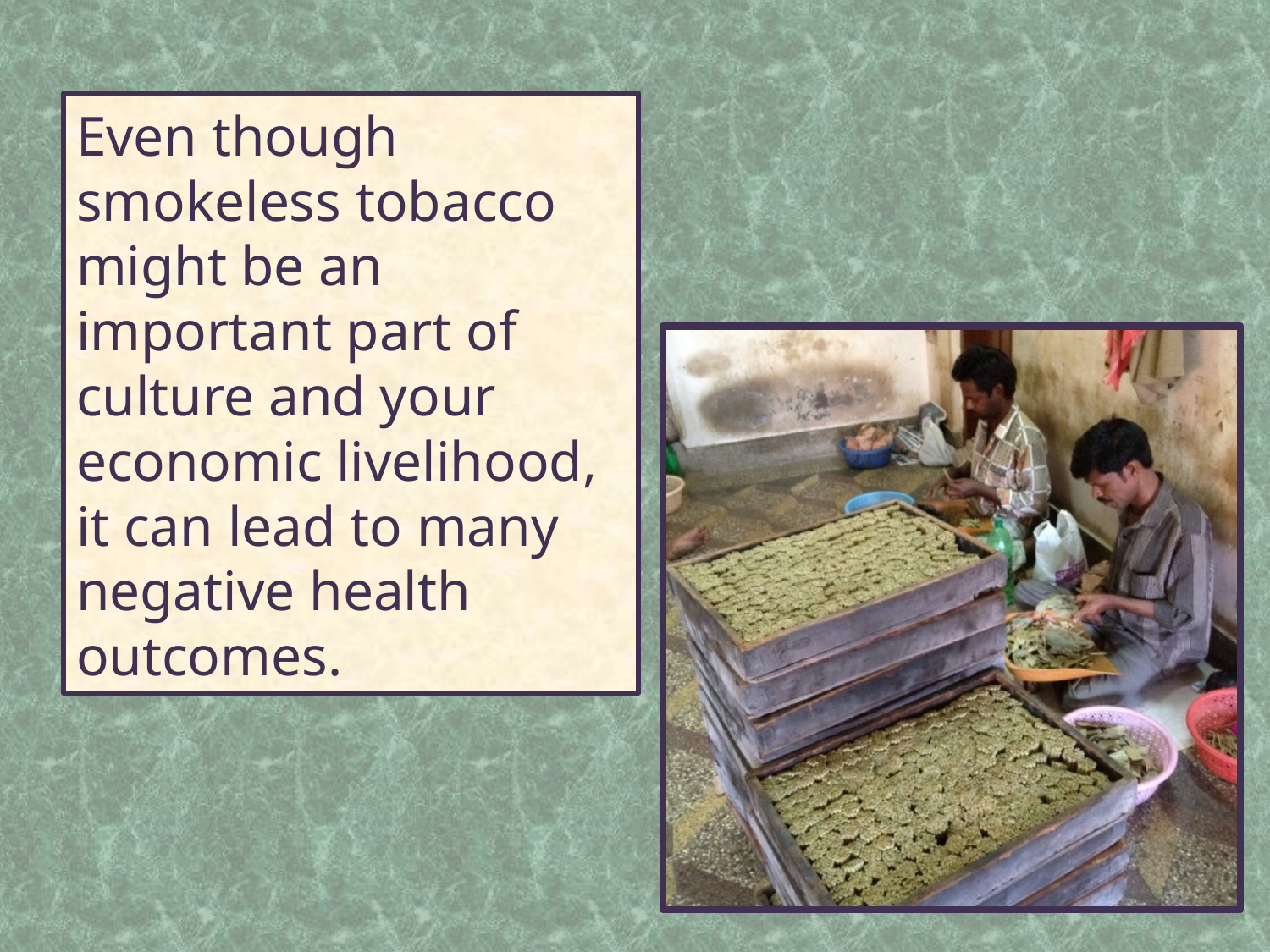

Even though smokeless tobacco might be an important part of culture and your economic livelihood, it can lead to many negative health outcomes.

## Slide 5
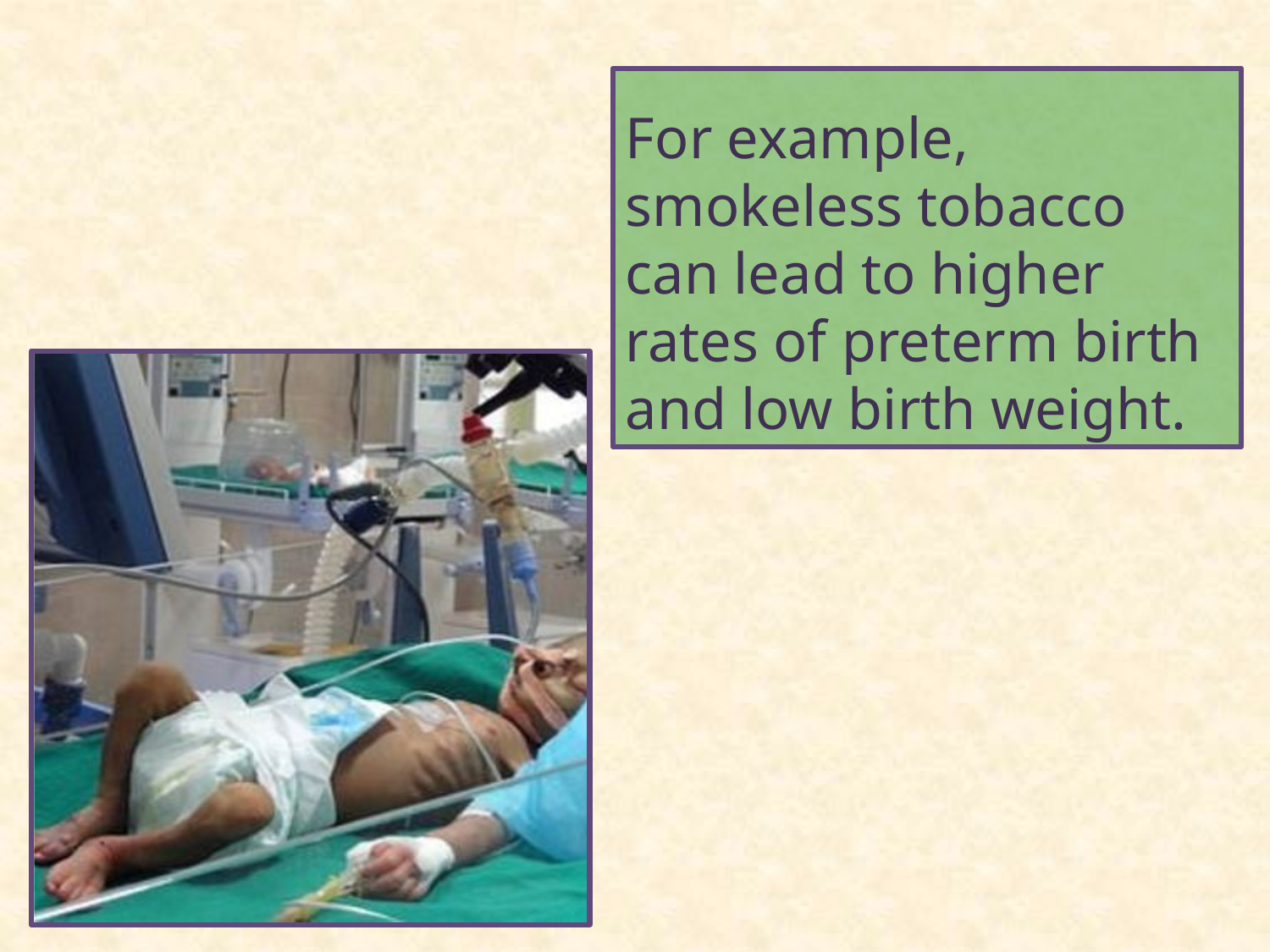

For example, smokeless tobacco can lead to higher rates of preterm birth and low birth weight.

## Slide 6
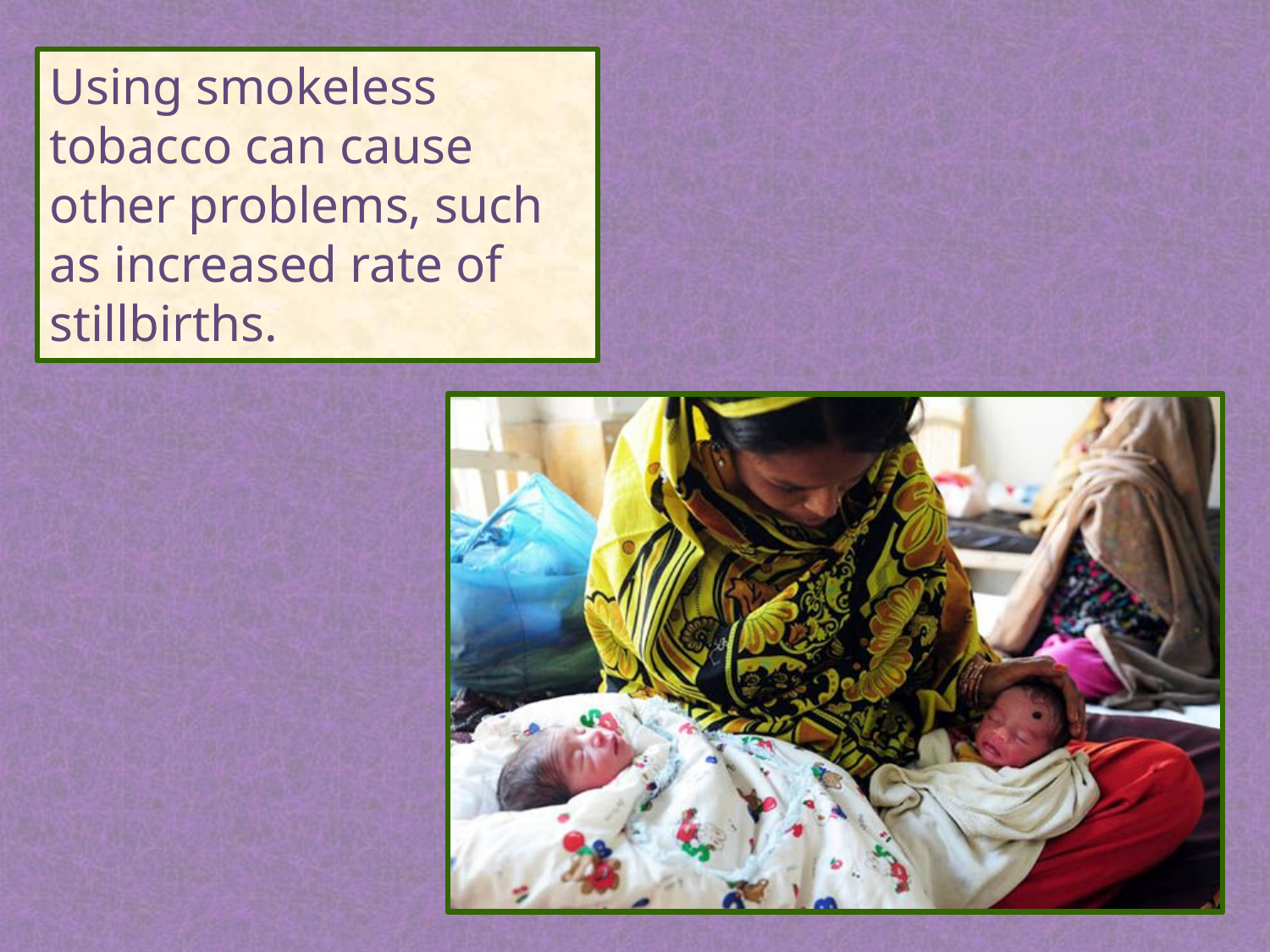

Using smokeless tobacco can cause other problems, such as increased rate of stillbirths.

## Slide 7
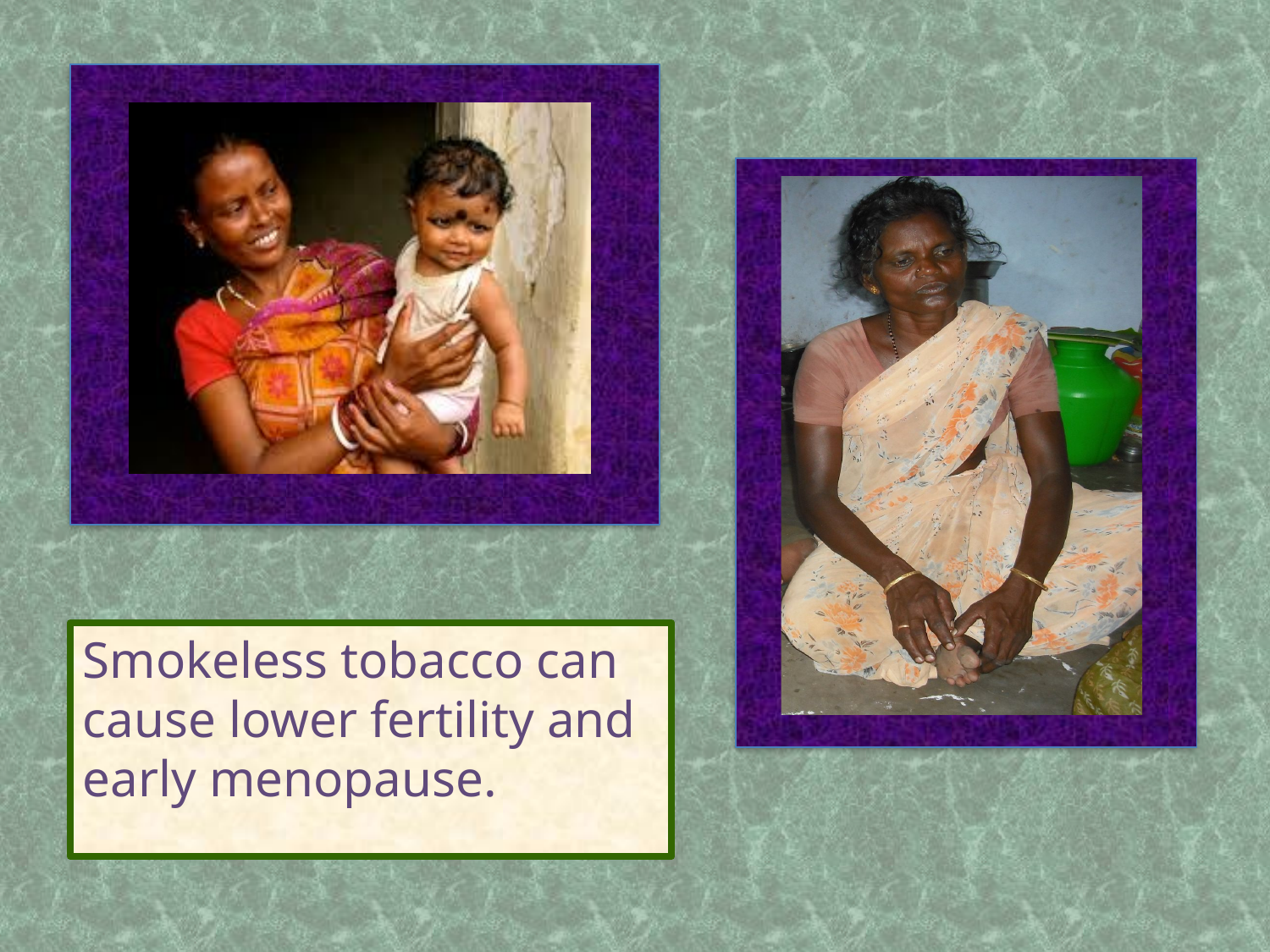

Smokeless tobacco can cause lower fertility and early menopause.

## Slide 8
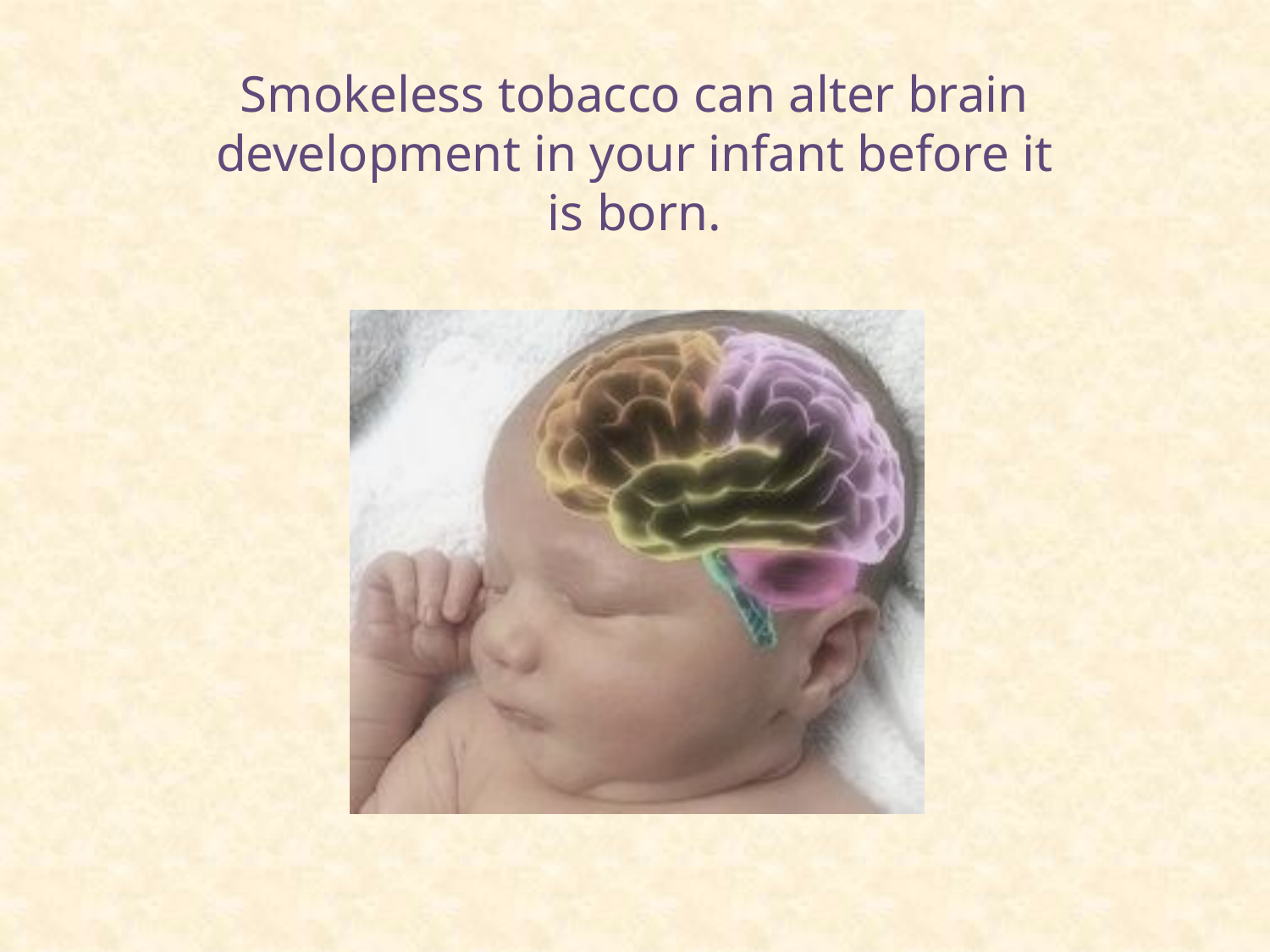

Smokeless tobacco can alter brain development in your infant before it is born.

## Slide 9
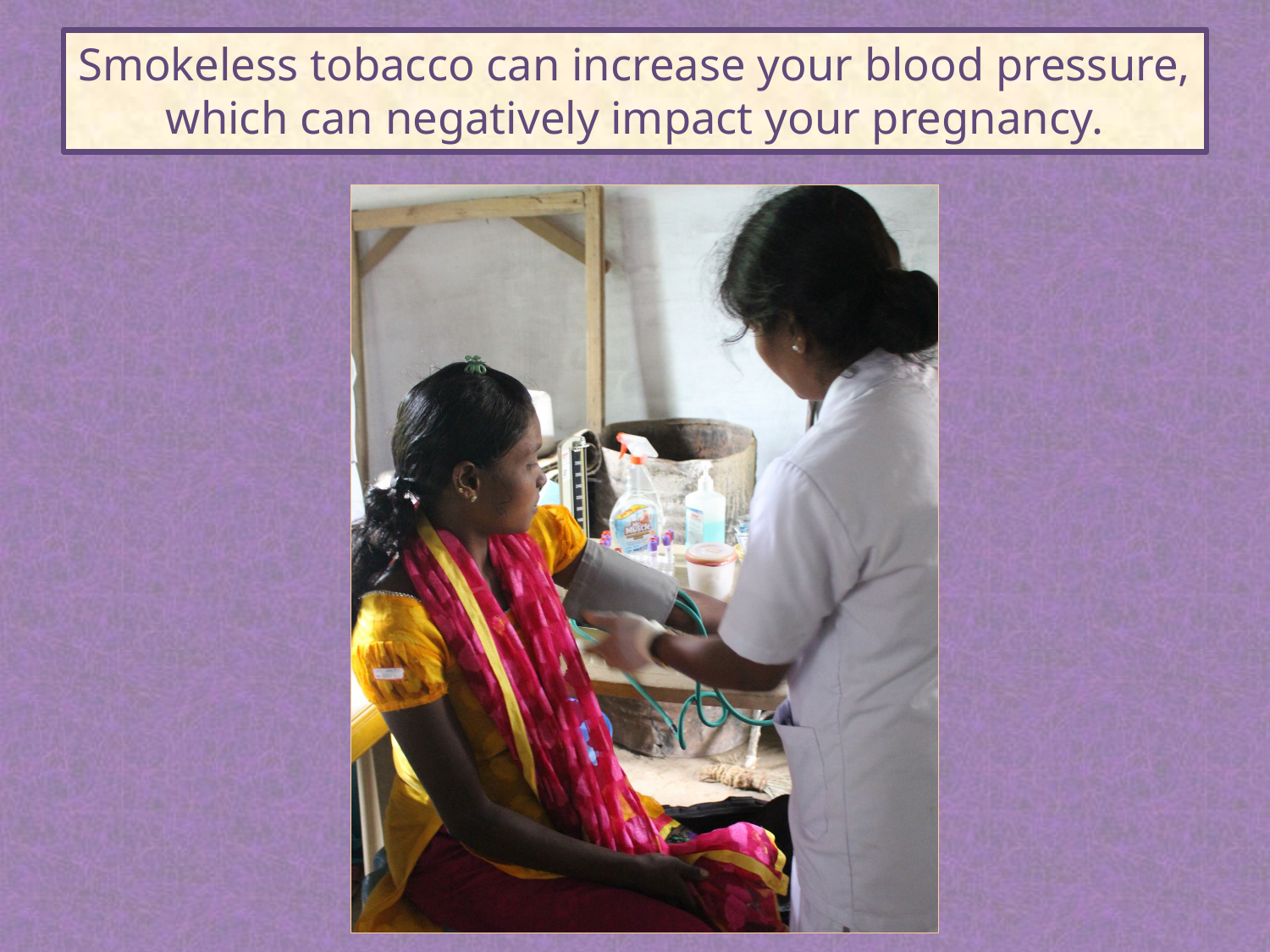

Smokeless tobacco can increase your blood pressure, which can negatively impact your pregnancy.

## Slide 10
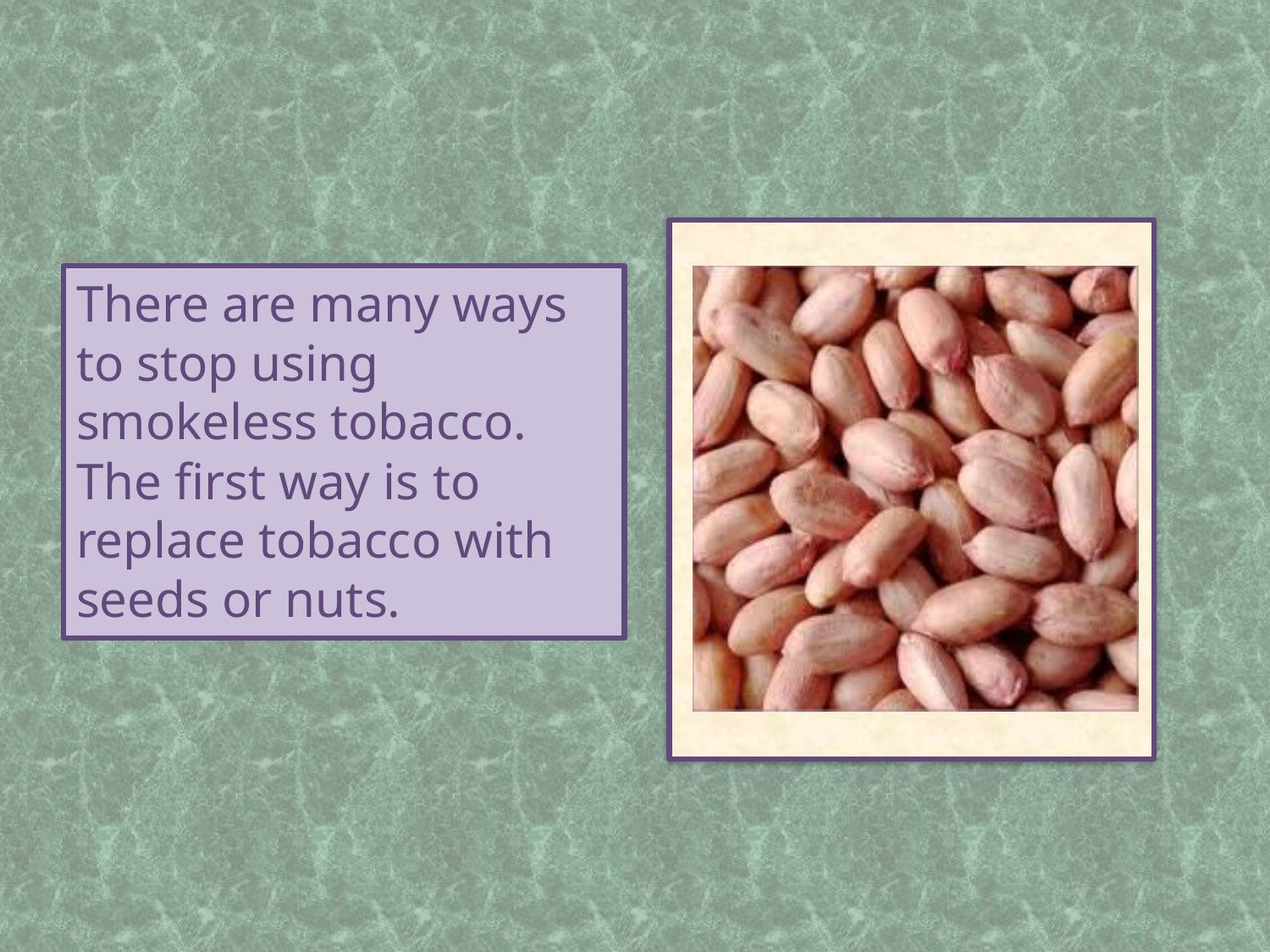

There are many ways to stop using smokeless tobacco. The first way is to replace tobacco with seeds or nuts.

## Slide 11
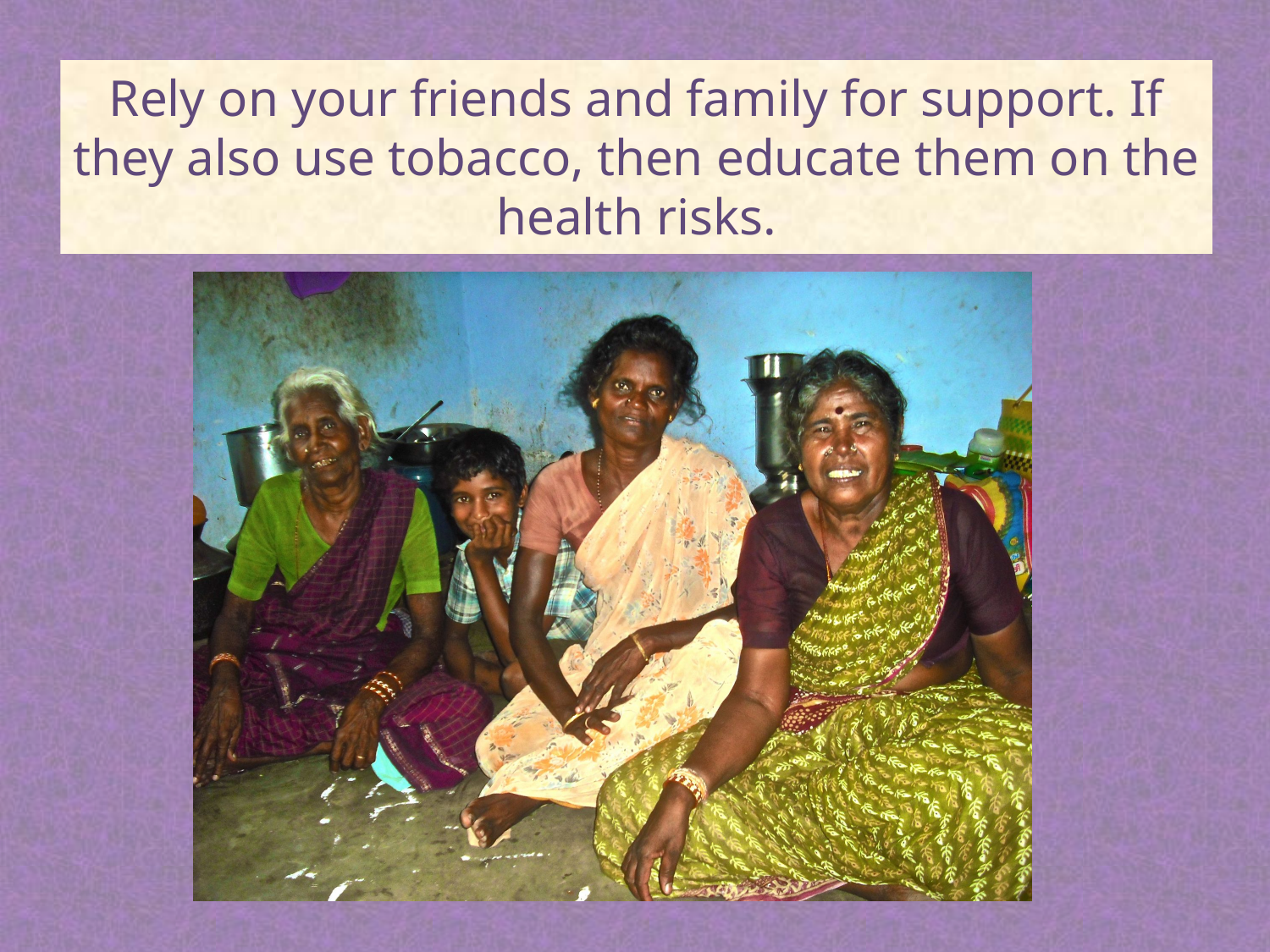

Rely on your friends and family for support. If they also use tobacco, then educate them on the health risks.

## Slide 12
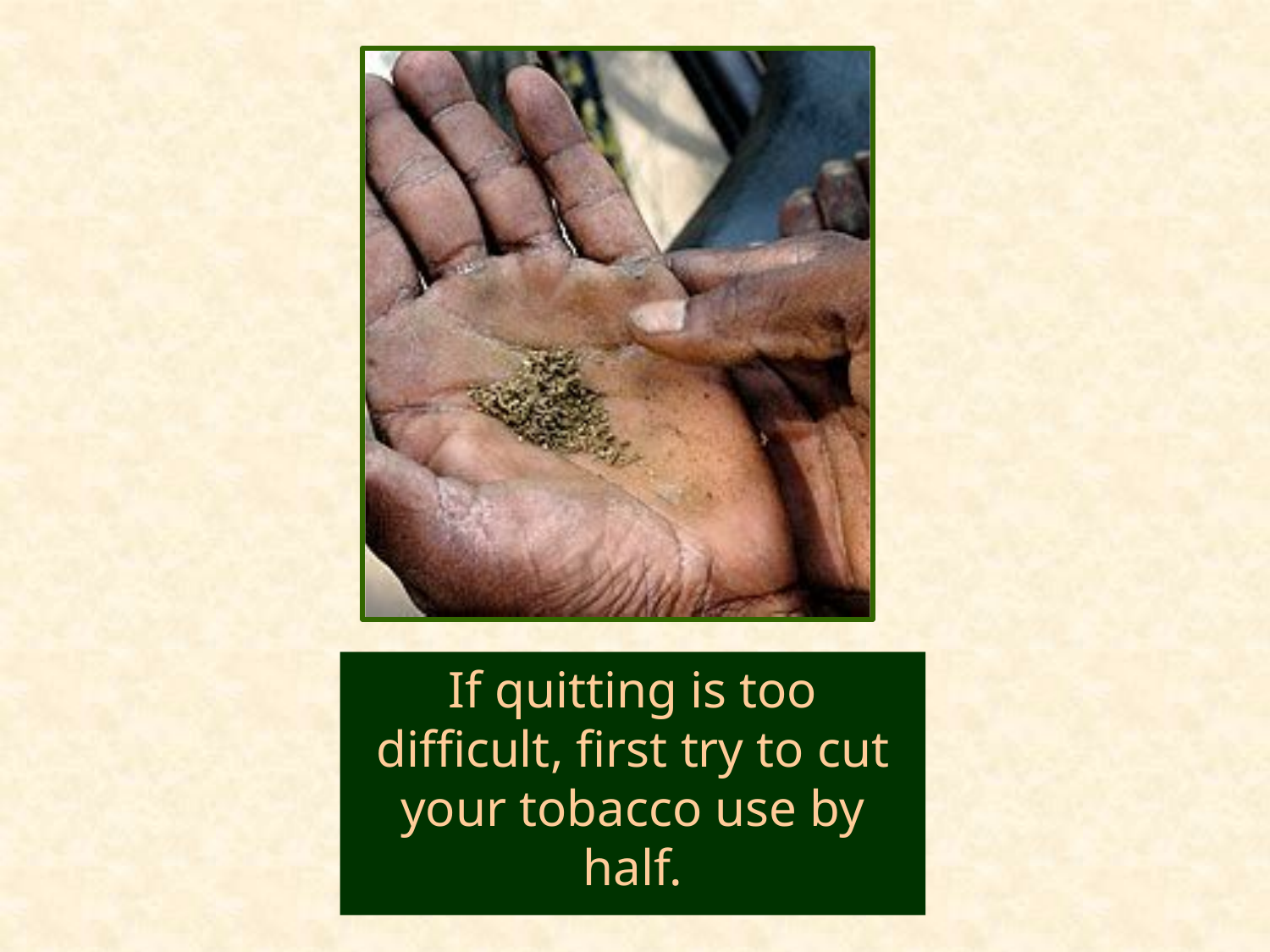

If quitting is too difficult, first try to cut your tobacco use by half.

## Slide 13
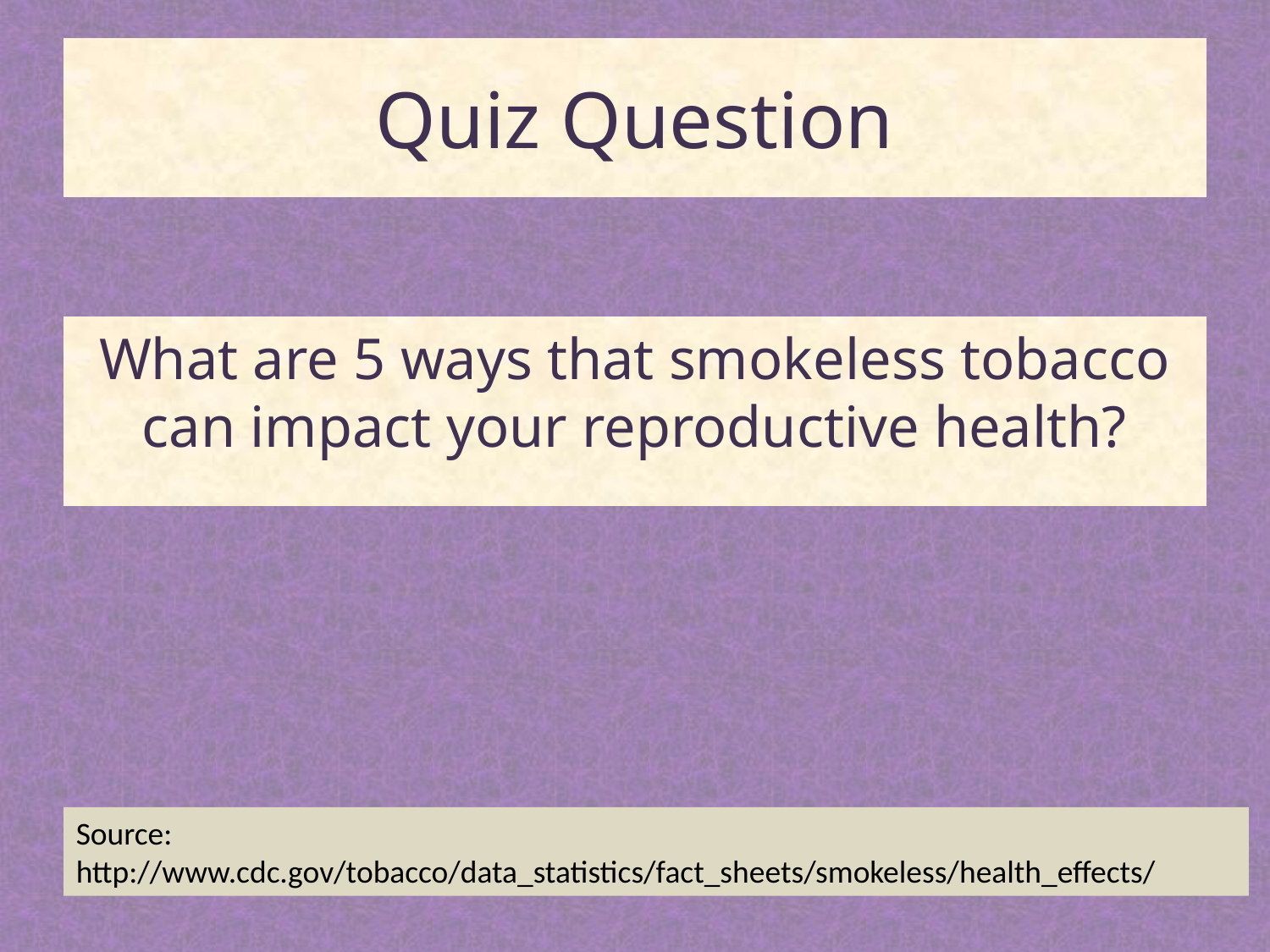

# Quiz Question
What are 5 ways that smokeless tobacco can impact your reproductive health?
Source:
http://www.cdc.gov/tobacco/data_statistics/fact_sheets/smokeless/health_effects/
