# Supplementary material for: The impact of information about tobacco-related reproductive vs. general health risks on South Indian women's tobacco use decisions
Source: Evol Hum Sci. 2020 Nov 20;3:e4. doi: 10.1017/ehs.2020.61 (PMC7996064; doi:10.1017/ehs.2020.61)
Supplement: Supplementary file 1 [file S2513843X20000614sup001.zip › Tobacco Study Questionnaire Pt. 1_3.30.docx]

**Tobacco Study Questionnaire**

**Women ages 14 & up**

**Demographic Questions**

1. Name:
2. Age:
3. Occupation:
   1. [If agriculture, list type of crops]
4. [If tobacco laborer] Describe what it is like to work in tobacco. E.g.: do you wear gloves or protective gear?
5. [If tobacco laborer] Do you ever harvest, cut, or load tobacco without gloves? If so, how often?
6. Have you ever gotten sick from harvesting tobacco? If so, how many times? If so, when was the last time? How long were you sick?
7. Do you store tobacco inside your house?
8. How many hours do you spend working each day?
   1. Domestic work:
   2. Non-domestic work:
9. Religion:
   1. Family god/goddess:
10. Caste:
11. Household monthly income:
12. Total years of education:
13. Family Status:
    1. Single
    2. Married
    3. Divorced
    4. Widowed
14. [If married]: Was your marriage arranged?
    1. Yes
    2. No
15. [If married]: How many children do you have?
16. How many children would you like to have?
17. [If married]: How many stillbirth deliveries have you experienced?
18. [If married]: How many miscarriages have you had?
19. [If married]: How many abortions have you had?
20. Are you currently suffering from any illnesses/diseases/allergies? If yes, what is it/are they?

**Pregnancy Questions [these questions were previously approved by IRB]**

1. How many months pregnant are you?
2. Are you currently experiencing nausea? Vomiting?
3. Are you currently experiencing any unusual cravings? If yes, please list the items.
4. Are you currently experiencing any aversions? If yes, please list the items.
5. Are there foods, beverages, or substances you avoiding at this stage in your pregnancy because someone told you to? Please list how you learned about which foods to avoid. And list the consequence(s) if you were to consume the item during pregnancy. [*If they list a disease/illness, ask them to describe it*].

**Tobacco Questions**

1. Do you currently use any type of tobacco?
   1. Yes
   2. No **[If no, skip to question 32]**
2. At what age did you start regularly using tobacco? (general age category is okay; e.g., childhood)
3. [If yes] What types of tobacco do you use?
4. [If yes] For each type, describe how you acquire it and how it is prepared.
5. [If yes] For each type of tobacco, state how often you used it in the last 24 hours.
6. [If yes to use] Does anyone know that you use ______ (fill in with type of tobacco)? If so, who?
7. How much money do you spend on tobacco each day?
8. Do you share your tobacco? If yes, with whom?
   1. Yes
   2. No
9. How much of your tobacco do you share each day?
10. Do any of your friends and family use tobacco?
    1. Yes (explain)
       1. Friends - who?
       2. Family – who?
       3. Both
    2. No
11. What types of tobacco do your friends and family use?
12. Do any of your friends or family pressure you to use tobacco?
13. Are there benefits to using tobacco? If yes, please list them:
14. Are there negative consequences to using tobacco? If yes, please list them:
15. Are there any negative consequences to quitting tobacco?
16. Have you ever seen any advertisements about using tobacco? If so, please describe the content that you recall seeing.
17. Is tobacco ever used as medicine? If yes, what ailments does tobacco cure?
18. Is tobacco ever used in any religious rituals? If yes, please describe.
19. Is it okay for women in your culture to use tobacco? Why or why not?
20. Do you currently use any other substances recreationally, such as marijuana, alcohol, or betel nut?
21. [If yes to question #45], describe how each substance is prepared.
22. [If yes to question #45], state how often you used it in the last 24 hours.

**Pathogen Exposure Scale [these questions were previously approved by IRB]**

1. In the last 7 days, have you: (HWWS= hand washed with soap)

HWWS after toilet? (yes/no)

HWWS before handling food? (yes/no)

1. How many people are consistently living in your household? (family that lives with you):
   1. How old is each person? (general age categories are okay)
   2. What is the sex of each person?
2. How many rooms are in your house?
3. What type of toilet do you have?
   1. None
   2. Pit
   3. Squat
4. On a scale from 1 to 10, how clean is the water that you usually drink? (0=not at all clean, 10= very clean)
5. How many animals live inside your house? Please list the type of animals.
6. How many animals surround your house? Please list the type of animals.

**Food Insecurity Scale [these questions were previously approved by IRB]**

1. In the last 12 months, since (date 12 months ago), did you (or other adults in your household) ever cut the size of your meals or skip meals because there wasn't enough money for food?
   1. Yes
   2. No
2. [Ask only if YES] How often did this happen-

a. Almost every month

b. Some months but not every month (OR)

c. In only 1 or 2 months?

1. In the last 12 months, did you ever eat less than you felt you should because there wasn't enough money to buy food?
   1. Yes
   2. No
2. In the last 12 months, since (*date 12 months ago*), were you ever hungry but didn't eat because you couldn't afford enough food?
   1. Yes
   2. No

*(Instructions: Now I'm going to read you 2 statements that people have made about their food situation. For these statements, please tell me whether the statement was often, sometimes, or never true for you [or the other members of your household] in the last 12 months.)*

1. The first statement is 'The food that [I/we] bought just didn't last, and [I/we] didn't have money to get more."

Was that often, sometimes, or never true for you in the last 12 months?

A. Often

B. Sometimes

C. Never

1. "[I/we] couldn't afford to eat balanced meals.' Was that often, sometimes, or never true for you in the last 12 months?
2. Often
3. Sometimes
4. Never

**Perceived infant mortality risk and adult mortality risk**

1. How likely are infants to live until the age of 1?
   1. Not likely
   2. Sometimes
   3. Very likely
2. How likely are infants to live until the age of 5?
   1. Not likely
   2. Sometimes
   3. Very likely
3. What are the major causes of stillbirths?
4. What are the major causes of death of adults in your community?
